# Structural investigation of heteroyohimbine alkaloid synthesis reveals active site elements that control stereoselectivity

**DOI:** 10.1038/ncomms12116

**Published:** 2016-07-15

**Authors:** Anna Stavrinides, Evangelos C. Tatsis, Lorenzo Caputi, Emilien Foureau, Clare E. M. Stevenson, David M. Lawson, Vincent Courdavault, Sarah E. O'Connor

**Affiliations:** 1 The John Innes Centre, Department of Biological Chemistry, Norwich NR4 7UH, UK; 2 Université François-Rabelais de Tours, EA2106 ‘Biomolécules et Biotechnologies Végétales', Tours 37200, France

## Abstract

Plants produce an enormous array of biologically active metabolites, often with stereochemical variations on the same molecular scaffold. These changes in stereochemistry dramatically impact biological activity. Notably, the stereoisomers of the heteroyohimbine alkaloids show diverse pharmacological activities. We reported a medium chain dehydrogenase/reductase (MDR) from *Catharanthus roseus* that catalyses formation of a heteroyohimbine isomer. Here we report the discovery of additional heteroyohimbine synthases (HYSs), one of which produces a mixture of diastereomers. The crystal structures for three HYSs have been solved, providing insight into the mechanism of reactivity and stereoselectivity, with mutation of one loop transforming product specificity. Localization and gene silencing experiments provide a basis for understanding the function of these enzymes *in vivo*. This work sets the stage to explore how MDRs evolved to generate structural and biological diversity in specialized plant metabolism and opens the possibility for metabolic engineering of new compounds based on this scaffold.

## Abstract (web summary)

The stereochemistry of the plant heteroyohimbine alkaloids is a key factor determining their diverse biological activities. Here, the authors carry out structural, localization and genetic experiments to understand the mechanism of stereoselectivity for three heteroyohimbine synthases and to identify their function *in vivo*.

## Graphical abstract

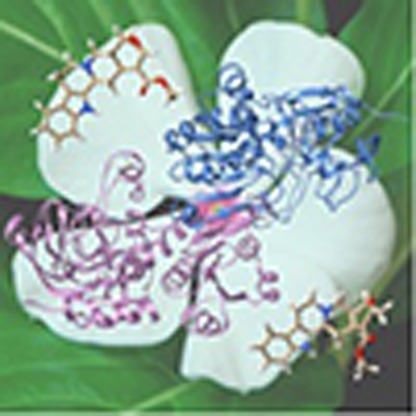

Heteroyohimbines are a prevalent subclass of the monoterpene indole alkaloids (Corynanthe type skeleton), having been isolated from many plant species, primarily from the Apocynaceae and Rubiaceae families^1^. These alkaloids exhibit numerous biological activities: ajmalicine is an α1-adrenergic receptor antagonist^2^,^3^,^4^,^5^, and mayumbine (19-epi-ajmalicine) is a ligand for the benzodiazepine receptor (Fig. 1)^6^. Oxidized beta-carboline heteroyohimbines also exhibit potent pharmacological activity: serpentine has shown topoisomerase inhibition activity^7^ and alstonine has been shown to interact with 5-HT2A/C receptors and shows promise as an anti-psychotic agent^8^,^9^,^10^,^11^,^12^,^13^. In addition, heteroyohimbines are biosynthetic precursors of many oxindole alkaloids, which also display a wide range of biological activities^14^. Although a total of 16 heteroyohimbine stereoisomers are possible, only 8 are reported to be found in nature, at stereocentres C3, C19, C20 (Fig. 1)^14^,^15^,^16^,^17^,^18^,^19^,^20^. How and why the stereoselectivity is controlled in the biosynthesis of these alkaloids remains unclear.

The medicinal plant *Catharanthus roseus* produces three of these isomers, ajmalicine (raubasine), tetrahydroalstonine and 19-epi-ajmalicine (mayumbine) (Fig. 1)^21^. These heteroyohimbines, along with the majority of monoterpene indole alkaloids, are derived from deglycosylated strictosidine (strictosidine aglycone)^22^. The removal of a glucose unit from strictosidine by strictosidine glucosidase (SGD) forms a reactive dialdehyde intermediate that can rearrange to form numerous isomers^23^. The stabilization of these isomers by enzyme-catalyzed reduction is hypothesized to be the stepping stone for the extensive chemical diversity observed in the monoterpene indole alkaloids (Fig. 1)^21^,^22^. We recently reported the first cloning of a biosynthetic gene encoding an enzyme that acts on strictosidine aglycone. This zinc-dependent medium chain dehydrogenase/reductase (MDR), named tetrahydroalstonine synthase (THAS), produces the heteroyohimbine tetrahydroalstonine (Fig. 1)^24^. Although these studies demonstrated that THAS is a key enzyme in heteroyohimbine biosynthesis, the mechanism by which this enzyme controls the stereoselectivity of the reduction remained unknown. Moreover, it is important to note that strictosidine aglycone serves as the precursor for many alkaloid scaffolds, and therefore represents a central branch point in the monoterpene indole alkaloid biosynthetic pathway. Therefore, we set out to identify additional heteroyohimbine synthases (HYSs) with different stereochemical product profiles that would more clearly define how structural diversity, in this case the formation of different stereoisomers, is controlled in this system.

In this study, we assayed 14 MDR homologues identified from the *C. roseus* transcriptome^25^,^26^ that have homology to THAS (Cr_024553). This screen revealed three additional enzymes with THAS activity (Cr_010119, Cr_021691, Cr_032583a), and, importantly, an enzyme that produced a mixture of heteroyohimbine diastereomers (Cr_032583b). Crystal structures of THAS (here referred to as THAS1), a second representative THAS (Cr_021691, THAS2) and the structure of the promiscuous homologue (Cr_032583b, HYS) were solved and mutants revealed key residues that control the stereochemistry of the product profiles. Notably, analysis of the subcellular localization of some of these HYSs indicates an unusual nuclear localization pattern and an interaction with the previous enzyme, SGD. These discoveries provide insight into the mechanism and evolution of a crucial branch point in a specialized metabolic pathway with both pharmacological and evolutionary importance.

## Results

### Discovery of HYSs

Guided by our initial discovery of THAS1 (ref. 24) we identified candidates from the MDR protein family in the *C. roseus* transcriptome^25^,^26^ based on amino acid similarity to this enzyme (Supplementary Table 1). Each of these candidates was cloned from *C. roseus* cDNA and expressed in *Escherichia coli*, with the exception of Cr_017994, which could not be expressed and was not considered further. The remaining candidates were assayed with the substrate strictosidine aglycone, and product formation was monitored by liquid chromatography mass spectrometry (LC-MS). Of these, four (Cr_010119, Cr_021691, Cr_032583a, Cr_032583b) reduced strictosidine aglycone to a product corresponding to one of the heteroyohimbines (Fig. 2, Supplementary Fig. 1). The products of the enzymatic reactions were identified based on LC-MS data and comparison to authentic standards (Supplementary Fig. 2). Enzymes that failed to produce a heteroyohimbine product were not studied further (Supplementary Fig. 1). Three of the enzymes (Cr_021691, THAS2; Cr_010119, THAS3; Cr_032583a, THAS4) produced tetrahydroalstonine in ∼85% yield, with small amounts of 19-epi-ajmalicine (mayumbine) (<15%) also observed in these reactions, similar to the previously reported THAS1. Notably, one enzyme (Cr_032583b, HYS) produced a dramatically different product profile consisting of a mixture of ajmalicine/tetrahydroalstonine/mayumbine (55:27:15, at pH 6) (Fig. 2). The discovery of this enzyme, HYS, now provides a molecular basis to understand the generation of stereochemical diversity in this alkaloid family.

### Crystallography of three HYSs

To understand the mechanism of stereochemical control at this crucial biosynthetic branch point, we crystallized three HYSs. THAS1 and THAS2, which produce predominantly tetrahydroalstonine, were both crystallized, since their amino acid sequence identity is relatively low (55%) and the predicted active sites have numerous differences (Fig. 3). HYS, which has a distinctly different product profile, was also crystallized to explore the structural basis behind this distinct stereochemical outcome. Structures (Supplementary Table 3) were obtained for THAS1 and THAS2 with NADP^+^ bound (THAS1, 1.05 Å resolution (Fig. 4a,b; Supplementary Figs 3–5); THAS2, 2.10 Å resolution (Fig. 4d, Supplementary Fig. 4)) and in apo form (THAS1, 2.25 Å resolution (Fig. 4c, Supplementary Fig. 5); THAS2, 2.05 Å resolution (Fig. 4c)), while HYS could only be crystallized in the apo form (2.25 Å resolution, Fig. 4c).

### Structural features of HYS active sites

The five HYS structures described here are similar to sinapyl alcohol dehydrogenase (SAD; PDB accession codes 1YQX and 1YQD) and the SAD homologue cinnamyl alcohol dehydrogenase (CAD; PDB accession codes 2CF5 and 2CF6)^27^,^28^,^29^, which reduce the aldehyde moiety of lignin precursors. Indeed, pairwise superpositions of subunits from these structures gave RMSD values of <2 Å (Supplementary Table 4). The biological unit is an elongated homodimer, with each subunit divided into a substrate and cofactor-binding domain; the latter also being responsible for forming the dimer interface (Supplementary Fig. 3). The overall structure of THAS1, with active site, cofactor and loops highlighted, is shown in Supplementary Fig. 3.

The active site cavities of the HYSs are framed by helix α2, the catalytic zinc coordination sphere, and loops 1 and 2, with the NADP(H) co-substrate binding at the base of the active site (Figs 3 and 4a,b,d,, Supplementary Fig. 4). Loop 2, which is positioned above the active site, is highly variable in length and sequence (Figs 3 and 4d). In both THAS1 and THAS2, a network of amino acids holds NADP^+^ in place. Most notably, Glu59 of THAS1 anchors NADP(H) through a bidentate interaction with both ribose hydroxyls, with His59 playing a comparable role in SAD, although here the interaction is with the 3′ OH only (Fig. 4b). Glu59 is conserved in HYS, but an aspartate residue (THAS3 and THAS2) or a tyrosine residue (THAS4) serves this role in other homologues. MDRs usually contain two zinc ions^30^, a distal ‘structural' zinc ion, which in this case is coordinated by four cysteine residues, and a proximal ‘catalytic' zinc ion near the active site, which is coordinated by two cysteines, one histidine and one glutamate residue (Figs 3 and 4b)^27^,^31^. The proximal zinc of THAS1 is ∼2 Å further away from the cofactor relative to SAD and thus may play no direct role in catalysis (Supplementary Fig. 4). However, it may have a function in maintaining the tertiary structure since three of the liganding residues are in the substrate-binding domain and the fourth is in the cofactor-binding domain. SAD/CAD utilize an active site serine that protonates the alkoxide that results from reduction of the aldehyde substrate^27^,^29^; this serine is replaced with a tyrosine residue in THAS1 (Tyr56) and HYS (Tyr53) (Fig. 3). In THAS2, this tyrosine on helix α2 is replaced with a tryptophan residue, but a tyrosine at position 120 points into the active site. Interestingly, a non-proline *cis*-peptide is present in the THAS1-NADP^+^ and HYS apo structures (Supplementary Fig. 5). Closer inspection of this region in THAS1 shows that when this bond is in the *trans* conformation, the side chain of Asp340 is projected into the cofactor-binding site such that it would prevent NADPH binding.

### Strictosidine aglycone binding

**Figure 1:**
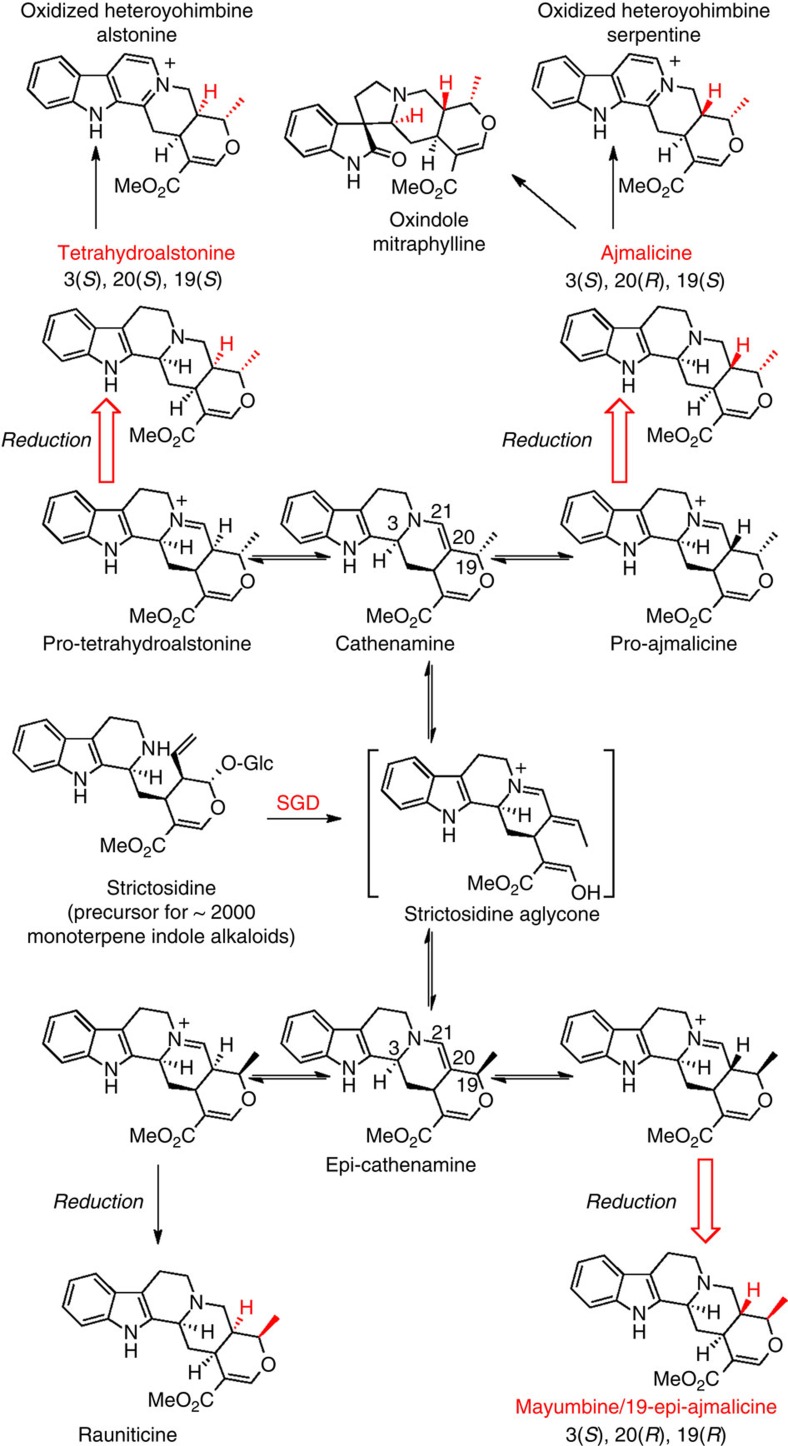
Heteroyohimbine alkaloid biosynthesis. Heteroyohimbines with 3(*S*) stereochemistry derive from strictosidine aglycone. The three diastereomers found in *Catharanthus roseus*, are highlighted with red arrows. Alkaloids derived from heteroyohimbines are also shown.

**Figure 2:**
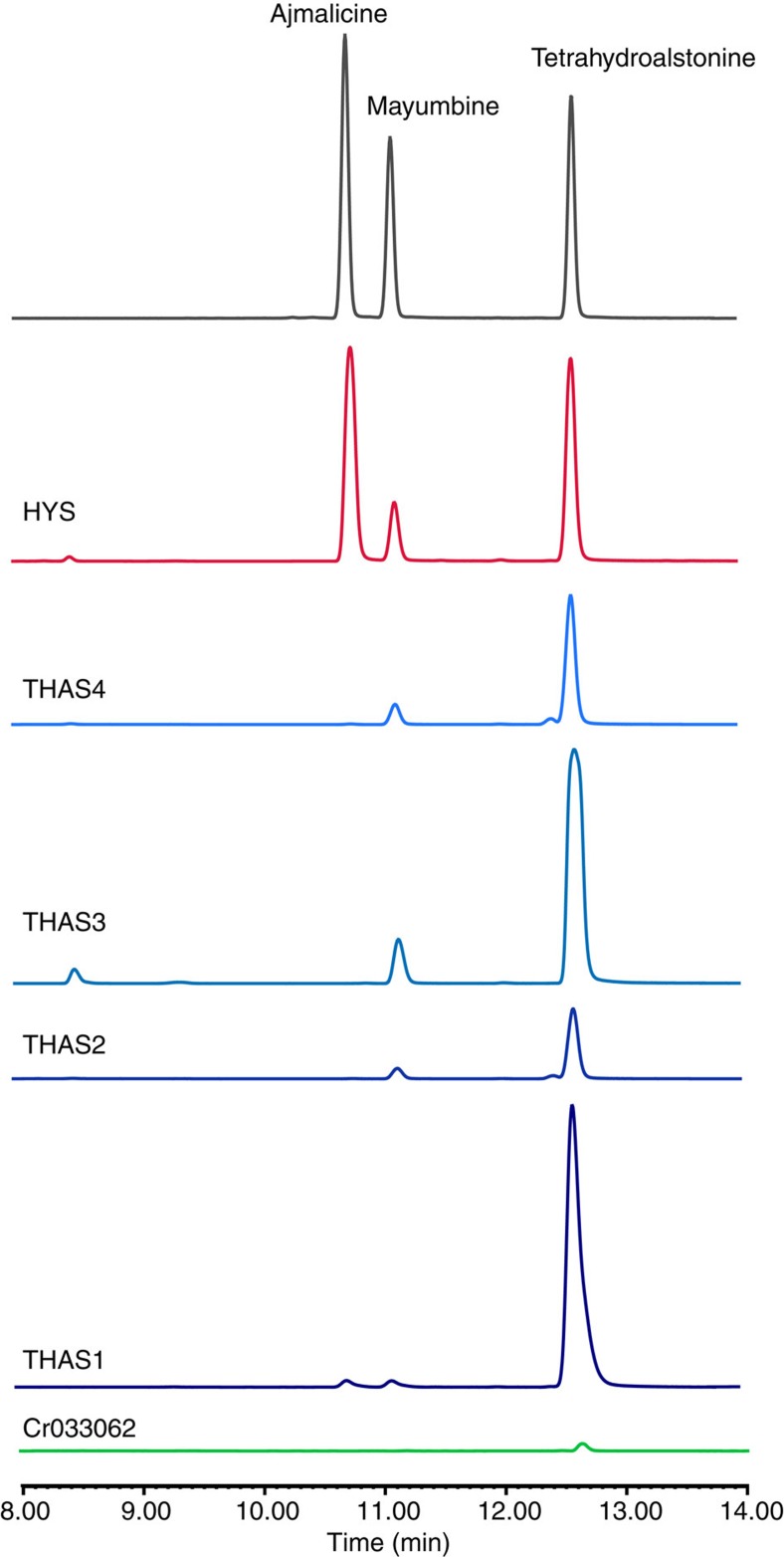
LC-MS analysis of active MDR candidates against strictosidine aglycone. Cr033062 exhibited only trace activity. See Supplementary Fig. 1 for chromatograms of assays with inactive enzymes and negative controls.

Despite extensive efforts, both product and substrate failed to co-crystallize with any of the enzymes. Therefore, molecular docking was used to visualize the position of strictosidine aglycone in THAS1 (Fig. 4b). To ensure that the correct substrate tautomer was used for docking, we identified the most predominant strictosidine aglycone isomer that forms in solution. Although product precipitation prevented monitoring the SGD reaction *in situ* under aqueous conditions (Methods), ^1^H,^15^N-HMBC NMR showed that an enamine species was the predominant product in aqueous methanolic solution (Supplementary Fig. 6). This is consistent with literature reports that cathenamine is the major product of SGD, and is the proposed precursor of ajmalicine and tetrahydroalstonine (Fig. 1)^21^,^23^. *In silico* docking with THAS1 positions cathenamine between the nicotinamide of the NADP^+^ and Tyr56, which is located on helix α2 (Fig. 4b). THAS1 loop 1 contains Phe65 that also projects into the active site and may interact with the aromatic cathenamine substrate.

### Mechanism of reduction and heteroyohimbine formation

In tetrahydroalstonine biosynthesis, we hypothesize that cathenamine tautomerizes to the iminium form by protonation at C20, followed by addition of the hydride at C21. Protonation at C20 must occur from the bottom face to yield the S stereochemistry observed at this position (Fig. 5a). While there does not appear to be an appropriately positioned active site residue to perform this role, the crystal structures of these enzymes reveal the presence of numerous water molecules in the active site that could potentially protonate this carbon (Fig. 4a). ^1^H,^15^N-HMBC measurements of strictosidine aglycone at different pH values show formation of the iminium species in solution when the pH was reduced to ∼3.5, indicating that this tautomer can readily form in the presence of an acidic moiety (Supplementary Fig. 6).

To elucidate the stereo and regioselectivity of reduction by NADPH, we isolated tetrahydroalstonine from reactions using THAS1 and pro-*R*-NADPD. Analysis by ^1^H-NMR showed that tetrahydroalstonine is labelled with deuterium in the pro-*R* position at C21, consistent with previously reported experiments performed in crude cell extracts (Fig. 6a)^32^. It is possible that THAS1 could reduce the enamine directly, in which case hydride addition would occur at C21, followed by protonation at C20 by a water molecule as described above. The presence of mayumbine/19-epi-ajmalicine in some of the enzymatic reactions suggests that small amounts of cathenamine can open and form 19-epi-cathenamine, either in solution or in the active site.

In the case of HYS, which produces both ajmalicine (*R* C20) and tetrahydroalstonine (*S* C20), protonation must also occur from the opposite face to yield *R* stereochemistry at C20 (Fig. 5b). Products of HYS generated with pro-*R*-NADPD were also isolated and analysed by ^1^H-NMR, and, as for tetrahydroalstonine from THAS1 in each case showed deuterium labelling in the pro-*R* position at C21 (Fig. 6a–c). Therefore, the stereochemical course of hydride addition is not altered in HYS compared with THAS1.

**Figure 3:**
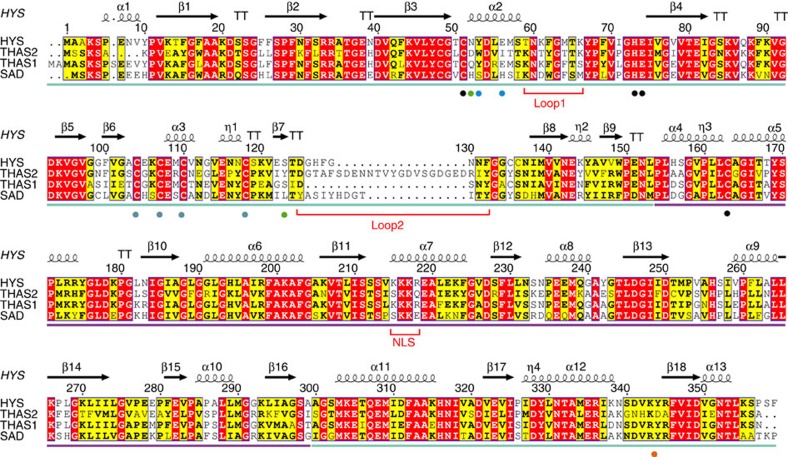
Sequence alignment of *Catharanthus roseus* HYS enzymes and *Populus tremuloides* SAD Numbering corresponds to HYS. Identical and similar amino acids are highlighted in red and yellow, respectively. Secondary structure elements of the HYS apo crystal structure are displayed. THAS1- and HYS-active site amino acids (Y56/53 and E59/56) are indicated by blue dots, and THAS2 active site amino acids (Y120 and D49) are indicated by green dots. Ligands for catalytic and structural zinc ions are highlighted by black and grey dots, respectively. The nuclear localization signal of (THAS1 and HYS) and loops 1 and 2, respectively, are indicated in red. A non-proline *cis*-peptide bond that is observed in THAS1 holo, in one subunit of THAS1 apo (Supplementary Fig. 5), in HYS apo, and not at all in THAS2 is indicated with an orange dot. The substrate-binding domain and the cofactor-binding domain are indicated by blue and purple bars, respectively.

The major difference between HYS and THAS1/THAS2 appears to be the extended loop over the HYS active site (D125-GHFGNN-F132 in HYS and D128-SN-Y131 in THAS1, loop 2 in Fig. 3). The histidine residue in HYS loop 2 (His127) appears to be positioned appropriately to provide an alternative proton source for the opposite (‘top') face of the substrate, which could explain the appearance of ajmalicine in the product profile of HYS. Reactions with THAS1 and HYS performed at different pH conditions (5–8) revealed that while changes in pH did not substantially impact the product profile of THAS1, HYS produced increased amounts of ajmalicine relative to tetrahydroalstonine at pH 6 compared with higher pH values (Supplementary Fig. 7). The increased level of ajmalicine in HYS at lower pH values is consistent with the pKa value of the histidine side chain and supports the role of this histidine in ajmalicine biosynthesis, though attributing pH dependence to specific residues must be approached with caution^33^.

### Switching stereoselectivity of HYSs

Since the major sequence and structural difference between HYS and THAS1 is the extended loop over the HYS active site, loop 2 (Fig. 3), we swapped these loop regions in THAS1 and HYS to determine whether the stereochemical product profile could be switched (Fig. 7a). Loop 1, which is near the active site, was also swapped (Fig. 7a). While the THAS1 mutant containing the swaps displayed reduced activity rather than an altered product profile (Fig. 7c), the HYS mutant containing the shorter THAS1 loop 2 resulted in a product profile similar to that of THAS1 (Fig. 7b, Supplementary Fig. 8). Since His127 is the only ionizable residue in this loop, we hypothesized that this residue protonates C20, as discussed above. Therefore, we mutated this histidine to alanine or asparagine in HYS. Both of these mutants gave the same THAS-like profile, suggesting that His127 is required for producing the ajmalicine (*R* C20) stereochemistry (Supplementary Fig. 9). Mutation of other conserved ionizable residues in the THAS1 active site (Tyr56, Ser102 and Thr166) did not result in substantial changes in the distribution of products (Supplementary Table 6, Supplementary Figs 10 and 11). Mutations to Glu59, which anchors the NADPH cofactor, resulted in a slight increase in product promiscuity (Supplementary Fig. 11), perhaps by causing a shift in the cofactor position. The reactivity of the substrate^34^ and precipitation at high concentrations during the assay makes obtaining accurate kinetic constants challenging^24^, so end-point assays were used to assess stereoselectivity and relative activity of the mutant enzymes (Supplementary Table 6). However, the *k*_cat_ (observed) values could be measured for THAS1 (1.518±0.059 s^−1^), THAS2 (0.033±0.001 s^−1^), THAS3 (0.102±0.004 s^−1^), THAS4 (0.044±0.006 s^−1^), HYS (0.083±0.005 s^−1^), HYS_loop2 swap (1.970±0.153 s^−1^), THAS1 Y56S (0.118±0.005 s^−1^) and THAS1 E59A (0.061±0.005 s^−1^).

### *In planta* localization of HYSs

Plants use spatial organization on the organ, tissue and intracellular levels to control product distribution (Supplementary Fig. 12). At the subcellular level, we previously showed that THAS1 has an unusual nuclear localization pattern^24^, which is also where SGD, the enzyme that synthesizes strictosidine aglycone, is localized^34^. Physical interactions using Bimolecular Fluorescence Complementation (BiFC) were observed between these two enzymes^24^. THAS2 and HYS localization were similarly investigated by expressing yellow fluorescent protein (YFP) fusions in *C. roseus* cells. Microscopy of transiently transformed cells revealed that THAS2–YFP (Fig. 8a) was located in both the cytosol and the nucleus while HYS–YFP (Fig. 8e), similar to THAS1, displayed a preferential nuclear localization (Fig. 8 and Supplementary Fig. 13). As reported for THAS1, this localization relies on the presence of a class V nuclear localization sequence in HYS (215-KKKR-218) that is absent from THAS2 (Fig. 3). BiFC assays revealed that both THAS2 and HYS are capable of self-interactions (Supplementary Fig. 14).

BiFC assays were used to determine whether THAS2 and HYS also interact with SGD (Fig. 9). C-terminal split-YFP fragment fusions of both enzymes (THAS2–YFPC and HYS–YFPC) were co-transformed with SGD that was fused to a N-terminal split-YFP fragment (YFPN–SGD). The formation of a nuclear BiFC complex suggests that both of these enzymes interact with SGD in the nucleus (Fig. 9a–d). Interestingly, the emitted fluorescent signal exhibited a punctated, sickle-shaped aspect as previously observed for the THAS1/SGD interaction (Fig. 9e,f) and for SGD localization^34^. In contrast, no interactions were detected when the BiFC assay was conducted with a downstream enzyme from this biosynthetic pathway, 16-hydroxytabersonine 16-*O*-methyltransferase (16OMT), that is not expected to interact with SGD (Fig. 9g,h).

Double BiFC assays were performed to combine the study of THAS2 and HYS interactions, as well as their interactions with SGD. After transformation into plant cells (16 h), we noted the formation of a dual fluorescent signal for THAS2, both in the cytosol and as punctates in the nucleus that may correspond to the superposition of the signal observed for THAS2 self-interactions and THAS2–SGD interaction (Fig. 9i,j) as confirmed by multicolour BiFC (mBIFC) assays (Supplementary Fig. 15). Increased time of expression (36 h) progressively resulted in the disappearance of the cytosolic signal, and it is intriguing to speculate that this implies a recruitment of THAS2 by SGD (Fig. 9q,r). A similar phenomenon was observed for HYS and THAS1 (Fig. 9k–n,s–v- Supplementary Fig. 15). While self-interactions of 16OMT were detected, no nuclear signal was recovered, confirming the specificity of THAS1–, THAS2–, HYS–SGD interactions (Fig. 9o,p).

### *In planta* silencing of HYSs

The expression levels of the HYS transcripts do not suggest whether a specific HYS is the most biologically relevant (Supplementary Fig. 12). To establish whether any of these enzymes synthesize the expected metabolic product *in planta* the genes encoding active HYSs were silenced. For many medicinal plants, including *C. roseus*, virus-induced gene silencing (VIGS) is the only established method to silence genes in the whole plant. In *C. roseus*, the effect of VIGS is temporally and spatially limited to the first two leaves that emerge immediately after infection^35^. Each of the genes encoding a biochemically active enzyme (*THAS1, THAS2, THAS3, THAS4* and *HYS*) was subjected to VIGS in *C. roseus* seedlings and the effect on alkaloid production was monitored by MS. Since *HYS* and *THAS4* were too similar to silence separately, one common gene fragment was used for silencing both genes simultaneously. Successful silencing of the genes was confirmed by quantitative reverse transcription–PCR (qRT–PCR) (Supplementary Fig. 16). Aside from a small degree of cross-silencing between *THAS2* and THAS3 (12%), all of the target genes were silenced selectively, as measured by qRT–PCR (Supplementary Fig. 16).

Due to the inherent reactivity of the HYS substrate (strictosidine aglycone), changes in the level of this compound *in planta* are difficult to accurately detect. Instead, the effect of silencing must be established by quantitatively measuring decreases in heteroyohimbine levels. Previously for *THAS1*, we measured the combined peak for heteroyohimbines, since the diastereomers were difficult to resolve on a reverse phase LC column under the reported conditions^24^. However, after substantial optimization (see Methods), an LC-MS method was developed to separate ajmalicine and tetrahydroalstonine in crude leaf extracts (19-epi-ajamlicine/mayumbine is not observed in *C. roseus* leaves, Supplementary Fig. 17). Ajmalicine and particularly tetrahydroalstonine are observed in low levels even in empty vector control samples compared with the major leaf monoterpene indole alkaloids vindoline and catharanthine, and in addition, heteroyohimbine composition varied substantially among individual leaves. Therefore, accurate measurement of decreases in ajmalicine and tetrahydroalstonine levels is challenging. There was no evidence for a decrease of ajmalicine or tetrahydroalstonine when *THAS2* and *THAS3* were silenced. However, for *HYS*, there was a statistically significant decrease (*t*-test 0.0275) in ajmalicine, and no change in tetrahydroalstonine levels. Surprisingly, a statistically significant decrease in ajmalicine, as well as tetrahydroalstonine (*t*-test 0.0277 and 0.0276, respectively) was also noted for *THAS1* (Supplementary Fig. 16). While the results are statistically significant, the leaf-to-leaf variability, the low level of endogenous production, and the catalytic redundancy of these enzymes make it difficult to draw firm conclusions from these VIGS data. In addition, regulatory factors that impact the ratio of ajmalicine and tetrahydroalstonine, such as transport mechanisms and/or further derivatization to other products, cannot be ruled out. Additional silencing systems, in different tissues, will be required to more firmly establish the physiological function of these enzymes. Nevertheless, we can state that silencing of *HYS* and *THAS1* impacts alkaloid production in *C. roseus* leaves.

## Discussion

Here we report several medium chain dehydrogenases/reductases that produce the heteroyohimbine stereoisomers ajmalicine and/or tetrahydroalstonine, thereby providing a framework to understand the enzymatic control over stereoselectivity in this metabolic pathway. It is notable that we have identified four enzymes that generate tetrahydroalstonine, yet ajmalicine is the more abundant isomer *in planta* (Supplementary Fig. 17). Expression profile data of the genes identified in this study suggest that HYS, which produces ajmalicine, is not expressed at higher levels than the other synthases (Supplementary Fig. 12). There may be additional ajmalicine synthases that are not related to the MDR superfamily homologues identified in this study. Alternatively, tetrahydroalstonine could be shuttled into another pathway or degraded, thereby resulting in the observed lower levels that accumulate *in planta*.

Importantly, the pharmacological activity of heteroyohimbines is impacted by the stereochemistry. Ajmalicine has recently been used in combination with almitrine in post-stroke treatments, though the side effects caused by almitrine resulted in widespread withdrawal of the drug in 2013 (ref. 4). While tetrahydroalstonine has no reported pharmacological function, its oxidized product, alstonine (Fig. 1), has recently been shown to act by a unique mechanism for modulating dopamine uptake and shows potential as an anti-psychotic drug^13^. The heteroyohimbines have excellent promise as a scaffold for pharmacological activity. The discovery of the HYSs, along with recently developed heterologous production platforms for monoterpene indole alkaloids^36^, now allows the possibility of generating these alkaloids and unnatural derivatives through metabolic engineering/synthetic biology strategies.

The crystal structures of three HYSs reveal the potential of biosynthetic machinery to generate stereochemical variation. Flexible loop regions can be the key to unlocking chemical diversity: as we have demonstrated here, mutating the extended loop over the HYS active site (loop 2 in Fig. 3) impacts stereochemical outcome. Notably, the MDRs that we have identified demonstrate high variability at this region (Fig. 3). Phylogenetic analysis (Supplementary Fig. 18) suggests that these HYSs, which appear to have originated from a common ancestor, may have undergone neo-functionalization through mutation in this loop region. This loop could potentially be harnessed in protein engineering efforts to generate novel catalytic activity.

While the *in planta* function of heteroyohimbines is unknown, deglycosylated strictosidine is toxic and may act as a defense compound^34^, similar to the defense roles of the aglycones of the iridoids from which stictosidine is derived^37^,^38^. SGD is expressed in most tissues (Supplementary Fig. 12), suggesting that the plant must have evolved mechanisms to control the levels of the toxic strictosidine aglycone. In directed overflow metabolism, excess reactive intermediates are converted into non-reactive byproducts^39^. It is intriguing to speculate that monoterpene indole alkaloid biosynthesis may have initially arisen as a mechanism for handling overflow of strictosidine aglycone. The HYSs perform a single, chemically straightforward reduction reaction that immediately neutralizes the reactivity of strictosidine aglycone/cathenamine. The co-localization and interaction of THAS1, THAS2 and HYS with SGD reinforces the hypothesis of an evolutionary mechanism deployed by strictosidine-accumulating plants to manage the reactivity of the strictosidine aglycone. It also raises the question of a possible competition between HYSs for recruitment by SGD when distinct enzymes are co-expressed in the same tissue/cells. Whether the heteroyohimbines serve an active biological function in the plant, or whether they are simply the end product of directed overflow metabolism, or both, remains to be investigated. Regardless, it is clear that MDRs play an important role in the generation of a wide variety of chemical structures. Duplication of the evolutionary dehydrogenase ancestor may have given rise to multiple HYSs, along with MDRs with other biosynthetic activities, such as tabersonine-3-reductase that is involved in the biosynthesis of the anti-cancer alkaloid vinblastine (Supplementary Fig. 18)^40^.

## Methods

### Selection and cloning of candidate MDRs

The nucleotide and the protein sequences of THAS1 were subjected to a BLAST search against the *C. roseus* Sunstorm Apricot V1.0 Transcript sequences (http://medicinalplantgenomics.msu.edu) and the MDR sequences with the highest identity to THAS1 at the active site and which showed non-negligible expression levels in young and mature leaves were selected as candidates for cloning and expression. The protein sequence of SAD was blasted against the same database and MDRs were also selected based on their active site similarity to that of SAD. The genes coding the candidate MDRs were amplified from *C. roseus* leaf cDNA and cloned into the *E. coli* expression vector pOPINF using the In-Fusion cloning kit (Clontech Takara)^41^ by using primers designed based on the transcript sequences (Supplementary Table 1).

### Site-directed mutagenesis of THAS1 and HYS

THAS1 mutants were generated by overlap extension PCR. Briefly, the codon to be mutated was selected and two primers, one reverse and one forward (Supplementary Table 4), were designed to overlap and introduce the mutation. A first PCR was carried out using the reverse mutant primer and the 5′ forward gene-specific primer (Supplementary Table 1), thus generating the 5′ half of the gene carrying the mutation. In parallel, the 3′ half of the mutated gene was generated by PCR using the forward mutant primer and the 3′ reverse gene-specific primer (Supplementary Table 4). PCR products were gel purified and used for the second PCR overlap reaction for generation of the full-length mutated gene, where the 5′ and 3′ halves of the mutated genes were mixed in a PCR reaction in equimolar amounts (∼100 ng per fragment) and five cycles of PCR were carried out without including primers. After the 5 overlap PCR cycles, the forward and reverse gene-specific primers were added to the mix and further 30 cycles were performed. Full-length PCR products were gel purified, ligated into pOPINF expression vector and transformed into competent *E. coli* Stellar strain cells (Clontech Takara). HYS point mutants were obtained as gene fragments (Integrated DNA Technologies, Belgium) with the H127 or F128 codons mutated (H127A CAT->GCA; H127N CAT->AAC; F128A TTT->GCT; F128Y TTT->TAC) and the pOPINF overhangs included at the 3′ and 5′ extremities. The THAS1 and HYS double loop mutants were generated by first making their loop 1 mutant genes and then inserting the second loop 2 swap following the same procedure described above. Mutant constructs were sequenced to verify the mutant gene sequence and correct insertion.

### Enzyme activity assays

All candidate enzymes and mutants were expressed in SoluBL21 (DE3) *E. coli* cells (Genlantis) grown in 2 × YT medium. Protein production was induced by addition of 0.2 mM IPTG and the cultures were shaken at 18 °C for 16 h. Cells were collected by centrifugation, lysed by sonication in Buffer A (50 mM Tris-HCl pH 8, 50 mM glycine, 500 mM NaCl, 5% v/v glycerol, 20 mM imidazole) supplemented with EDTA-free protease inhibitor (Roche Diagnostics) and 0.2 mg ml^−1^ lysozyme. Soluble proteins were purified on Ni-NTA agarose (Qiagen) and eluted with Buffer B (50 mM Tris-HCl pH 8, 50 mM glycine, 500 mM NaCl, 5% v/v glycerol, 500 mM imidazole). Eluates were analysed by SDS–PAGE to verify the purity and the molecular weight of the purified proteins. All proteins were dialysed in Buffer C (50 mM phosphate pH 7.6, 100 mM NaCl) and concentrated. Protein concentration was measured with Bradford reagent (Sigma-Aldrich) according to the manufacturer's instructions. Purified proteins were divided in 20 μl aliquots, fast-frozen in liquid nitrogen and stored at −20 °C.

Candidate MDR enzymes and the selected mutants were screened for activity against deglycosylated strictosidine. The substrate was generated by deglycosylating strictosidine (300 μM) by the addition of purified SGD in the presence of 50 mM phosphate buffer (pH 6.5) at room temperature for 10 min. The reactions were started by the addition of MDR enzyme (1 μM) and NADPH (5 mM). Caffeine (50 μM) was used as internal standard. All reactions were performed in triplicate. Aliquots of the reaction mixtures (10 μl) were sampled 1 and 30 min after addition of MDR enzyme. The reactions were stopped by the addition of 10 μl of 100% MeOH. Samples were diluted 1:5 in mobile phase (H_2_O+0.1% formic acid) and centrifuged for 10 min at 4,000*g* before UPLC-MS injection (1 μl). The activity of MDR enzymes and mutants was measured by UPLC-MS. Enzymes exhibited a loss of activity after one freeze thaw cycle.

The initial velocity was determined for the wild-type enzymes that displayed HYS activity (THAS1-4 and HYS), as well as for the mutants THAS1 Y56S, E59A and HYS loop 2 swap. Reactions were monitored at 340 nm at room temperature using a (Cary 50 Bio, Varian) spectrophotometer. Strictosidine (50 μM) was incubated with purified SGD in 50 mM phosphate buffer (pH 7.0) for ∼30 min at 30 °C. NADPH (100 μM) was added, mixed by pipetting and the absorbance at 340 nm was monitored until stabilized (minimum of 2 min). A predetermined amount of enzyme (10–400 nM) was then added, mixed and the reaction was monitored for a minimum of 5 min at 340 nm. The resulting slope was calculated during the linear reaction range, usually 0–60 s after addition of the enzyme. The reactions were replicated five times and the initial velocity was calculated for each replicate after accounting for the background. The concentration of substrate (50 μM strictosidine) was determined to be saturating for all wild-type enzymes under these conditions.

### Protein crystallization

Proteins for crystallization were purified from 2 l cultures in 2 × YT medium. Protein expression was induced by addition of IPTG and the cultures were grown for 16 h at 18 °C. Cells were collected by centrifugation and lysed by sonication in 50 ml Buffer A supplemented with EDTA-free protease inhibitor and 10 mg of Lysozyme. Lysates were clarified by centrifugation at 17,000*g* for 20 min. Two-dimensional automated purification was performed on an AKTAxpress purifier (GE Healthcare). The IMAC step was performed on HisTrap HP 5 ml columns (GE Healthcare) equilibrated with Buffer A. Proteins were step-eluted with Buffer B and directly injected on a gel filtration column equilibrated with Buffer D (20 mM HEPES, 150 mM NaCl, pH 7.5). Fractions were collected and analysed by SDS–PAGE and those containing pure protein were pooled and concentrated in a 10 kDa membrane filter—Millipore filter (Merck Millipore).

Purification of HYS required the addition of 1 mM DTT to all purification buffers and dialysis in Buffer D containing 0.5 mM *tris*(2-carboxyethyl)phosphine (TCEP) before crystallization and storage.

Crystallization screens were conducted by sitting-drop vapour diffusion in MRC2 96-well crystallization plates (Swissci) with a mixture of 0.3 μl well solution from the PEGs (Qiagen), PACT (Qiagen) and JCSG (Molecular Dimensions) suites and 0.3 μl protein solution. Protein concentrations were adjusted to 7–10 mg ml^−1^ while NADP^+^ (Sigma-Aldrich) was added to a final concentration of 1 mM for co-crystallization studies. Solutions were dispensed either by an OryxNano or an Oryx8 robot (Douglas Instruments).

THAS1 apo crystals were obtained from His_6_-tag cleaved THAS1 (3C protease) in a solution containing 0.1 M MES, pH 6.5, 15% w/v PEG 2000. THAS1-NADP^+^ crystals were obtained from a solution containing 0.2 M potassium/sodium tartrate with 20% w/v PEG 3350. THAS2 crystals (with and without NADP^+^) were obtained from a condition containing 0.2 M lithium chloride and 20% w/v PEG 3350. HYS crystals were obtained after removal of the His_6_-tag (using 3C protease) in 0.1 M MMT buffer, pH 5 and 15% w/v PEG 3350. All crystals were cryoprotected by soaking in crystallization solution containing 25% v/v ethylene glycol before flash-cooling in liquid nitrogen.

### Data collection and structure determination

**Figure 4:**
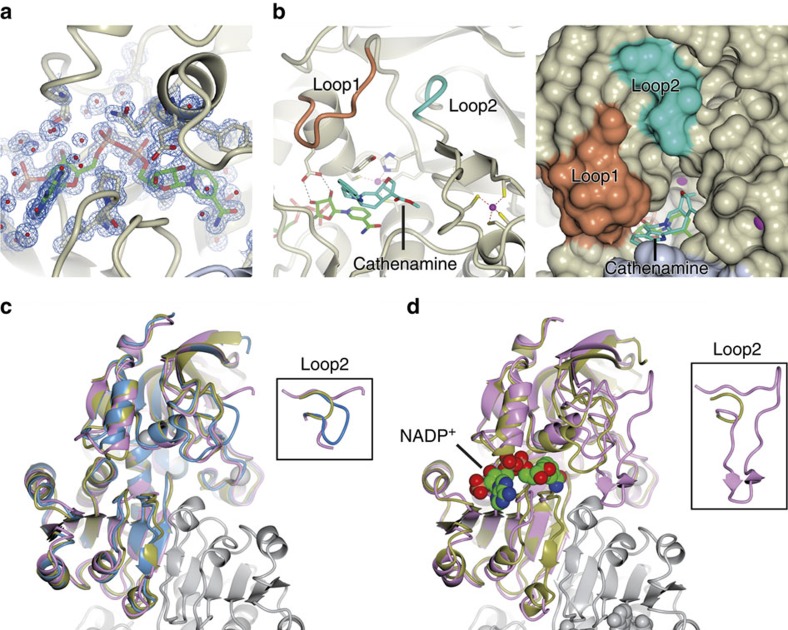
Crystal structures of heteroyohimbine synthases THAS1, THAS2 and HYS. (**a**) Sample of automatically derived experimentally phased electron density from THAS1 (at 1.12 Å resolution) superimposed on the final model showing the active site region with the NADP^+^ cofactor (green carbons) together with neighbouring residues (magnolia carbons) and water molecules (small red spheres). (**b**) THAS1 docked with cathenamine (pale blue carbons) with the protein shown in both cartoon (left) and space filling (right) modes. The NADP^+^ cofactor is shown with green carbons; loop 1 is in orange and loop 2 is in cyan. Zinc ions are displayed as magenta spheres. The active site is largely contained within a single subunit (magnolia surface), although the mouth of the channel leading to the active site is partially bounded by the second subunit of the biological dimer (grey surface) (**c**) Superposition of the apo structures of THAS1 (gold), THAS2 (pink) and HYS (blue). (**d**) Superposition of the holo (NADP^+^ containing) structures of THAS1 (gold) and THAS2 (pink), with the cofactor of THAS1 shown as van der Waals spheres for emphasis. For **c** and **d**, the structures were superposed onto the THAS1 structure, based on the upper subunit alone; only part of the lower subunit of the THAS1 structure is shown in grey for reference (see Supplementary Fig. 3 for images of the full THAS1 dimer). The insets emphasize the differing lengths of loop 2 between the various structures; the central portion of loop 2 in apo THAS2 was disordered.

**Figure 5:**
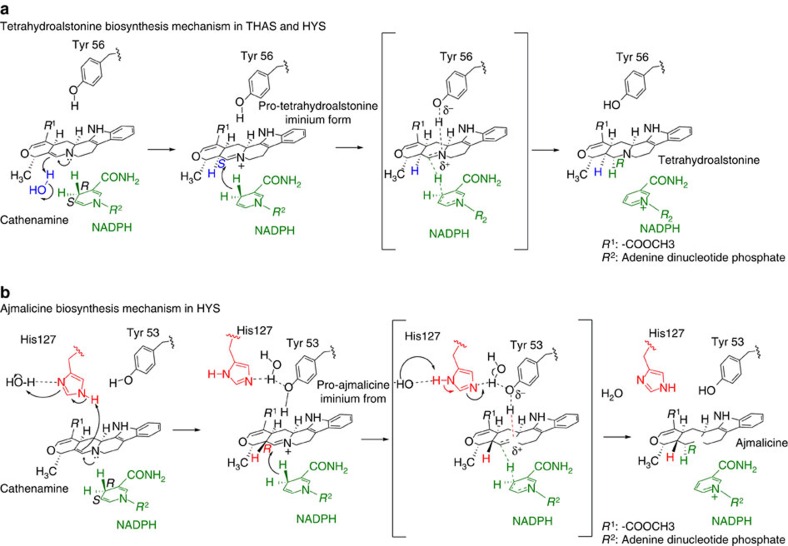
Mechanistic hypothesis for heteroyohimbine synthases. (**a**) Proposed mechanism for formation of the tetrahydroalstonine (*S* C20) diastereomer. (**b**) Proposed mechanism of formation of the ajmalicine (*R* C20) diastereomer that is observed in HYS, which contains a histidine residue near the active site.

X-ray data sets were recorded on one of three beamlines at the Diamond Light Source (Oxfordshire, UK) (2FI3, I04; 2FI5, I03; 5H81 I04-1; 5H82, I04-1; 5H83, I04-1) at wavelengths of 0.9000–0.976 Å (2FI3, 0.900 Å; 2FI5, 0.976 Å; 5H81, 0.920 Å; 5H82, 0.920 Å; 5H83, 0.920 Å) using either a Pilatus 6M or 2M detector (Dectris) with the crystals maintained at 100 K by a Cryojet cryocooler (Oxford Instruments). Diffraction data were integrated using XDS^42^ and scaled and merged using AIMLESS^43^ via the XIA2 expert system^44^; data collection statistics are summarized in Supplementary Table 3. Initially the THAS1-NADP^+^ data set was automatically processed at the beamline by fast_dp^45^ to 1.12 Å resolution and a structure solution was automatically obtained by single wavelength anomalous dispersion phasing using the SHELX suite^46^ via the fast_ep pipeline (Winter, manuscript in preparation). Despite being collected at a wavelength somewhat remote from the zinc *K* X-ray absorption edge (theoretical wavelength 1.284 Å), the anomalous signal was sufficient for fast_ep to locate four zinc sites and calculate a very clear experimentally phased electron density map (Fig. 4a). This was available to view at the beamline in the ISPyB database^47^ via the SynchWeb interface^48^ within a few minutes of completing the data collection. The map was of sufficient quality to enable 94% of the residues expected for a THAS1 homodimer to be automatically fitted using BUCCANEER^49^. The model was finalized by manual rebuilding in COOT^50^ and restrained refinement using anisotropic thermal parameters in REFMAC5 (ref. 51) against the same data set reprocessed to a resolution of 1.05 Å as described above (Supplementary Table 3), and contained 97% of the expected residues, with one NADP^+^ molecule and two zinc ions per subunit. All the remaining structures were solved by molecular replacement using PHASER.^52^ In each case, the asymmetric unit corresponded to the biological dimer and the preliminary models were obtained by searching for two copies of a monomer template. For THAS1 apo, THAS2 NADP^+^ and HYS apo, a THAS1-NADP^+^ protein-only monomer model was used as the basis for the template, although in the latter two cases a homology model of the target structure was generated from the THAS1 template using the Phyre2 server^53^ (http://www.sbg.bio.ic.ac.uk/~phyre2) before running PHASER. For solving the THAS2 apo structure, a THAS2 NADP^+^ monomer was used as the template. In contrast to THAS1-NADP^+^, these four structures were refined in REFMAC5 with isotropic thermal parameters and TLS group definitions obtained from the TLS-MD server^54^. Model geometries were validated with the MOLPROBITY^55^ tool before submission to the PDB. The statistics of the final models are summarized in Supplementary Table 3. Additional statistics for *R*_pim_: 5FI3, 0.020 (0.517); 5FI5, 0.038 (0.600); 5H81, 0.041 (0.349); 5H82, 0.033 (0.439); 5H83, 0.068 (0.664) and CC_½_: 2FI3, 0.999 (0.510); 2FI5, 0.999 (0.523); 5H81, 0.998 (0.725); 5H82, 0.999 (0.639); 5H83, 0.996 (0.510) (where values in parentheses are for highest-resolution shell) were also noted. Ramachandran statistics (favoured/allowed/outlier (%)) are 5FI3, 96.8/3.2/0.0; 5FI5, 96.0/4.0/0.0; 5H81, 96.2/3.8/0.0; 5H82, 96.1/3.9/0.0; 5H83, 96.6/3.1/0.3. All structural figures were prepared using CCP4mg^56^.

Simulated annealing omit maps were calculated for the active site regions of all five structures presented in this study. For all structures, selected residues bordering the active site (and the cofactors in the case of the two holoenzyme structures) were deleted from the coordinates of the final models. The resultant PDB files were used as inputs to simulated annealing refinement with PHENIX (https://www.phenix-online.org) from a starting temperature of 5,000 K after applying small random shifts to the model (‘shake' term set to 0.3). The resultant *mF*obs–*dF*calc difference electron density maps (contoured at ∼3.0*σ*) are displayed superposed on the final coordinates, where the corresponding omitted atoms are shown in stick representation. In each case, structurally equivalent residues were omitted, with the exception of HYS where His127 from loop 2 was also omitted.

### UPLC-MS analysis

Enzyme assays and plant tissue samples from VIGS experiments on *C. roseus* plants were analysed by UPLC-MS. UPLC-MS analysis was carried out on a UPLC (Waters) equipped with an Acquity BEH C18 1.7 μm 2.1 × 50 mm column connected to Xevo TQS (Waters). MS detection was performed in positive ESI. Capillary voltage was 3.0 kV; the source was kept at 150 °C; desolvation temperature was 500 °C; cone gas flow, 50 l h^−1^; and desolvation gas flow, 800 l h^−1^. Unit resolution was applied to each quadrupole. Multiple reaction monitoring signals were used for detection and quantification of caffeine (*m/z* 195>110, 138) and heteroyohimbine alkaloids (353>117, 14).

For rapid dereplication of active enzymes and mutants, a linear gradient method (Method 1) was used at a flow rate of 0.6 ml min^−1^ using a binary solvent system in which solvent A1 was 0.1% formic acid in water and solvent B1 was acetonitrile. The gradient profile was: 0 min, 5% B1; from 0 to 3.5 min, linear gradient to 35% B1; from 3.5 to 3.75 min, linear gradient to 100% B1; from 3.75 to 4 min, wash at 100% B1; back to the initial conditions of 5% B1 and equilibration for 1 min before the next injection. Column temperature was held at 30 °C. The injection volume for both the solutions of standard compounds and the samples was 1 μl. Samples were kept at 10 °C during the analysis.

For separation of the different heteroyohimbines, a different chromatographic method was applied that was adapted from the work of Sun J. *et al*.^57^ In this method (Method 2) solvent A2 was 0.1% NH_4_OH and solvent B2 was 0.1% NH_4_OH in acetonitrile. A linear gradient from 0 to 65% B2 in 17.5 min was applied for separation of the compounds followed by an increase to 100% B2 at 18 min, a 2-min wash step and a re-equilibration at 0% B2 for 3 min before the next injection. The column was kept at 60 °C throughout the analysis and the flow rate was 0.6 ml min^−1^.

### ^2^H labelling experiments

Deuterated Pro-*R*-NADPD was regenerated in solution by *Thermoanaerobacter brockii* alcohol dehydrogenase (50 U, Sigma) using 400 μM NADP^+^ and 1% v/v [^2^H_6_]-isopropanol (CIL). The NADPD regeneration was monitored by ultraviolet spectroscopy at 340 nm. Strictosidine (19.9 mg) was incubated with 1.27 nM SGD in 94 ml of 50 mM phosphate buffer (pH 6.5). THAS1 enzyme was added to the reaction (final concentration of 1.65 μM) and the mixture was incubated at 35 °C. The reaction was monitored for completeness by UPLC-MS and after 5 h no strictosidine or deglycosylated strictosidine was observed. The reaction was stopped by addition of 100 ml of methanol and reaction mixture was concentrated to dryness. The dried reaction mixture was resuspended in 15 ml H_2_O and extracted with 3 × 15 ml of ethyl acetate and the EtOAc fraction was dried. [21α-^2^H_1_]-tetrahydroalstonine was isolated by preparative TLC (nano-silica plate, Sigma-Aldrich), as previously described^24^. The band of [21α-^2^H_1_]-tetrahydroalstonine was excised from the plate and THA was extracted with EtOAc multiple times (total volume 40 ml). The EtOAc fraction was filtered and dried under high-vacuum overnight. The [21α-^2^H_1_]-tetrahydroalstonine was dissolved in 600 μl of CDCl_3_ and ^1^H-NMR was measured.

Strictosidine (39.3 mg) was incubated with 1 nM of SGD and 500 μM NADP^+^ with 50 U of *T. brockii* ADH and 1% v/v [^2^H_6_]-isopropanol in a total volume of 148 ml of 50 mM HEPES buffer (pH 7.5). HYS was added (final concentration 1.71 μM) and the reaction was incubated at 37 °C with shaking and monitored for completeness by UPLC-MS. After 6 h, the reaction was complete and was stopped by addition of 150 ml of methanol. [21α-^2^H_1_]-tetrahydroalstonine, [21α-^2^H_1_]-ajmalicine and [21α-^2^H_1_]-mayumbine were isolated by preparative TLC and ^1^H-NMR spectra measured as described above.

### ^15^N labelling experiments

*C. roseus* tryptophan decarboxylase (TDC) was cloned into pOPINF vector, expressed in *E. coli* and purified as described above for the MDRs. [alpha-^15^N]-tryptophan (CIL, 50 mg) was incubated with 500 nM of TDC, 400 μM pyridoxal-5′-phosphate in 100 ml 50 mM phosphate buffer (pH 7.5) at 35 °C. The reaction was monitored by MS, continued through completion after 4 h and terminated by addition of 50 ml MeOH. [alpha-^15^N]-tryptamine (34 mg) was isolated by preparative HPLC. The isolated [alpha-^15^N]-tryptamine was incubated with 3 mM secologanin and 200 nM strictosidine synthase in 100 ml 50 mM phosphate buffer (pH 7.0) at 30 °C overnight. The reaction was terminated by addition of 50 ml MeOH. [4-^15 ^N]-strictosidine (62 mg) was isolated by preparative HPLC. [4-^15^N]-strictosidine was then assayed with SGD and the product was extracted the ethyl acetate, dissolved in MeOH-*d*_4_ and characterized by ^1^H,^15^N-HMBC as described above.

### Compound characterization

High-resolution electrospray ionization MS spectra were measured with a Shimadzu IT-TOF mass spectrometer. NMR spectra were acquired using a Bruker Advance NMR instrument operating at 400 MHz for ^1^H equipped with a BBFO plus 5-mm probe. The number of scans depended on the concentration of the sample. The ^1^H,^15^N-HMBC experiment was acquired with a spectral width 6,009 Hz in the F2 (^1^H) dimension and 30,410 Hz in the F1 (^15^N), with an acquisition time of 0.09 s and 360 scans per increment. The long range delay was optimized after a series of experiments with [4-^15^N]-strictosidine using a range of different mixing times and finally was adjusted for a coupling of 5 Hz. The relaxation delay was 2.5 s, the data collection matrix was 1024 × 64, the t1 dimension was zero filled to 1k real data points and a *π*/2 square sine bell window was applied in both dimensions. The ^1^H-NMR spectra were compared with those of standards and literature data.

*[21α*-^*2*^*H*_*1*_*]-tetrahydroalstonine.* TLC (EtOAc: C_6_H_14_: Et_3_N, 24: 75: 1 v/v): *R*_f_=0.58; HR-MS (IT-TOF): found for [M+H]+: [C_21_H_24_DN_2_O_3_]^+^=354.1921: calcd 354.19225; ^1^H-NMR (400 MHz, CDCl_3_): δ 7.80 (br s, 1H), 7.56 (s, 1H), 7.45 (dd, *J*=7.6 Hz, *J*=1.8 Hz, 1H), 7.28 (dd, *J*=7.6 Hz, *J*=1.8 Hz, 1H), 7.12 (ddd, *J*=7.6 Hz, *J*=7.6 Hz, *J*=1.8 Hz, 1H), 7.07 (ddd, *J*=7.6 Hz, *J*=7.6 Hz, *J*=1.8 Hz, 1H), 4.50 (dq, *J*=10.4 Hz, *J*=6.3 Hz, 1H), 3.74 (s, 3H), 3.36 (dd, *J*=12.0 Hz, *J*=3.0 Hz, 1H), 3.08 (d, *J*=2.0 Hz, 1H), 2.95 (ddd, *J*=12.2 Hz, *J*=6.1 Hz, *J*=2.5 Hz, 1H), 2.89 (ddd, *J*=14.3 Hz, *J*=6.1 Hz, *J*=2.5 Hz, 1H), 2.77 (ddd, *J*=12.0 Hz, *J*=4.5 Hz, *J*=3.0 Hz, 1H), 2.69 (ddd, *J*=14.3 Hz, *J*=10.7 Hz, *J*=3.6 Hz, 1H), 2.56 (ddd, *J*=12.2 Hz, *J*=10.4 Hz, *J*=4.8 Hz, 1H), 2.49 (ddd, *J*=12.0 Hz, *J*=4.5 Hz, *J*=3.0 Hz, 1H). 1.70 (ddd, *J*=10.4 Hz, *J*=4.7 Hz, *J*=2.0 Hz, 1H), 1.53 (dd, *J*=12.0 Hz, *J*=12.0 Hz, 1 H), 1.40 (d, 6.3, 3H).

*[21α*-^*2*^*H*_*1*_*]-ajmalicine*. TLC (EtOAc: C_6_H_14_: Et_3_N, 24: 75: 1 v/v): *R*_f_=0.41; HR-MS (IT-TOF): found for [M+H]+: [C_21_H_24_DN_2_O_3_]+=354.1921: calcd 354.19225; ^1^H-NMR (400 MHz, CDCl_3_): δ 7.94 (br s, 1H), 7.53 (d, *J*=1.8 Hz 1H), 7.46 (dd, *J*=7.7 Hz, *J*=1.5 Hz, 1H), 7.30 (dd, *J*=7.7 Hz, *J*=1.5 Hz, 1H), 7.13 (ddd, *J*=7.7 Hz, *J*=7.7 Hz, *J*=1.5 Hz, 1H), 7.08 (ddd, *J*=7.7 Hz, *J*=7.7 Hz, *J*=1.5 Hz, 1H), 4.43 (dq, *J*=13.3 Hz, *J*=6.7 Hz, 1H), 3.74 (s, 3H), 3.41 (ddd, *J*=11.3 Hz, *J*=4.3 Hz, *J*=1.8 Hz 1H), 3.22 (m, 1H), 3.10 (m, 1H), 3.00 (m, 1H), 2.95 (d, *J*=3.1 Hz, 1H), 2.75 (m, 1H), 2.68 (m, 1H), 2.42 (m, 1H), 2.14 (ddd, *J*=13.3 Hz, *J*=11.4 Hz, *J*=3.1 Hz, 1H), 1.32 (dd, *J*=11.4 Hz, *J*=11.4 Hz, 1H), 1.19 (d, 6.7, 3H).

*[21α*-^*2*^*H*_*1*_*]-mayumbine*. TLC (EtOAc: C_6_H_14_: Et_3_N, 24: 75: 1 v/v): *R*_f_=0.45; HR-MS (IT-TOF): found for [M+H]^+^: [C_21_H_24_DN_2_O_3_]^+^=354.1921: calcd 354.19225; ^1^H-NMR (400 MHz, CDCl_3_): δ 7.96 (br s, 1H), 7.56 (d, *J*=1.8 Hz 1H), 7.46 (dd, *J*=7.6 Hz, *J*=1.6 Hz, 1H), 7.30 (dd, *J*=7.6 Hz, *J*=1.6 Hz, 1H), 7.13 (ddd, *J*=7.6 Hz, *J*=7.6 Hz, *J*=1.6 Hz, 1H), 7.09 (ddd, *J*=7.6 Hz, *J*=7.6 Hz, *J*=1.6 Hz, 1H), 3.88 (dq, *J*=12.0 Hz, *J*=6.2 Hz, 1H), 3.73 (s, 3H), 3.40 (dd, *J*=11.1 Hz, *J*=4.4 Hz, 1H), 3.16 (ddd, *J*=12.6, Hz *J*=3.0 Hz, *J*=3.0 Hz, 1H), 3.14 (dd, *J*=12.0 Hz, *J*=6.0 Hz, 1H), 3.10 (d, *J*=3.1 Hz, 1H), 3.03 (ddd, *J*=14.8 Hz, *J*=10.9 Hz, *J*=2.5 Hz, 1H), 2.72 (dd, *J*=16.3 Hz, *J*=6.7 Hz, 1H), 2.40 (ddd, *J*=13.8 Hz, *J*=10.5 Hz, *J*=3.4 Hz, 1H), 1.76 (ddd, *J*=10.3 Hz, *J*=10.3 Hz, *J*=3.3 Hz, 1H), 1.37 (d, 6.3, 3H), 1.30 (dd, *J*=14.6 Hz, *J*=11.0 Hz, 1H), 1.21 (dd, *J*=16.2 Hz, *J*=6.7 Hz, 1 H).

*[4*-^*15*^*N]-strictosidine*. HR-MS (IT-TOF): found for [M+H]^+^: [C_27_H_35_N^15^NO_9_]+=532.2304: calcd 532.23074; ^1^H-NMR (400 MHz, CD_3_OD): δ 10.5 (br s, 1H), 7.77 (s, 1H), 7.44 (dd, *J*=7.8 Hz, *J*=1.9 Hz, 1H), 7.30 (dd, *J*=8.1 Hz, *J*=1.6 Hz, 1H), 7.11 (ddd, *J*=8.1 Hz, *J*=7.0 Hz, *J*=1.9 Hz, 1H), 7.02 (ddd, *J*=7.8 Hz, *J*=7.0 Hz, *J*=1.6 Hz, 1H), 5.84 (ddd, *J*=17.5 Hz, *J*=10.4 Hz, *J*=7.5 Hz, 1H), 5.83 (d, *J*=9.0 Hz, 1H), 5.34 (dd, *J*=17.4 Hz, *J*=2.7 Hz, 1H), 5.26 (dd, *J*=10.6 Hz, *J*=2.6 Hz, 1H), 4.79 (d, *J*=7.9 Hz, 1H), 3.96 (dd *J*=12.0 Hz, *J*=2.2 Hz, 1H), 3.78 (s, 3H), 3.64 (dd, *J*=11.8 Hz, *J*=6.9 Hz, 1H), 3.42–3.30 (m, 3H), 3.23 (dd, *J*=9.8 Hz, *J*=8.9 Hz, 1H), 3.22 (dd, *J*=9.3 Hz, *J*=7.9 Hz, 1H), 3.10-3.00 (m, 2H), 2.97 (ddd, *J*=15.9 Hz, *J*=4.9 Hz, *J*=1.3 Hz, 1H), 2.72 (ddd, *J*=8.5 Hz, *J*=7.9 Hz, *J*=4.6 Hz, 1H), 2.27 (dd, *J*=14.6 Hz, *J*=11.5, 1H), 2.18 (dd, *J*=11.4 Hz, *J*=3.8, 1H); ^15^N-NMR (40 MHz, CD_3_OD): δ 44.8.

### Subcellular localizations and analysis of protein–protein interactions by BiFC

Subcellular localization of THAS2 and HYS were studied by creating fluorescent fusion proteins using the pSCA-cassette YFPi plasmid^58^. The full-length open reading frame of THAS2 was amplified using the specific primers 5′-CTGAGAACTAGTATGTCTTCAAAATCAGCAAAACCAGTG-3′ and 5′-CTGAGAACTAGTAGCAGATTTCAATGTGTTTTCTATGTCAAT-3′, and HYS ORF with primers 5′-CTGAGAACTAGTATGGCTGCAAAGTCACCTGAAAATGTATAC-3′ and 5′-CTGAGAACTAGTGAAAGATGGGGATTTGAGAGTGTTTCCTAC-3′, which were designed to introduce the *Spe*I restriction site at both cDNA extremities. PCR products were sequenced and cloned at the 5′ end of the YFP-coding sequence to generate the THAS2–YFP, HYS–YFP fusion proteins or at the 3′ end to express the YFP–THAS2 and YFP–HYS fusions.

The interaction of THAS2 and HYS with SGD were characterized by BiFC assays using the previously amplified THAS2 and HYS PCR products cloned via *Spe*I into the pSPYCE (M) vector^34^, which allows expression of THAS2 and HYS fused to the amino-terminal extremity of the split-YFP^C^ fragment (THAS2–YFP^C^, HYS–YFP^C^, respectively), and into the pSPYNE(*R*)173-SGD plasmid^34^ expressing SGD fused to the carboxy-terminal extremity of the split YFP^N^ fragment (YFP^N^–SGD). Plasmids encoding THAS1–YFP^N^, THAS1–YFP^C^, YFP^C^–THAS1 and plasmids expressing 16OMT–YFP^N^ and 16OMT–YFP^C^ were used as controls and were constructed previously^24^,^59^.

THAS2 and HYS self-interactions were analysed via additional cloning of the THAS2 and HYS PCR products into the pSCA-SPYNE173, pSPYNE(*R*)173 and pSCA-SPYCE (MR) plasmids^34^,^60^ to express THAS2–YFP^N^, HYS–YFP^C^ and YFP^C^–THAS2, YFP^C^–HYS, respectively.

The capacities of THAS2 and HYS to interact with SGD were also characterized by double BiFC and mBIFC. The previously amplified THAS2 and HYS PCR products were fused to the coding sequences of the amino-terminal or carboxy-terminal of the split YFP fragments into the pSCA-SPYNE173, pSCA-SPYCE (M) and pSCA-SPYCE (MR) plasmids^34^,^60^, allowing expression of THAS2–YFP^N^, YFP^C^–THAS2 HYS–YFP^N^ and HYS–YFP^C^ respectively. SGD was subsequently fused to the carboxy-terminal extremity of the split YFP^N^ fragment (YFP^N^–SGD) and the CFP^N^ fragment (CFP^N^-SGD).

Transient transformation of *C. roseus* cells by particle bombardment and fluorescence imaging were performed following the procedures previously described^58^. Briefly, *C. roseus*-plated cells were bombarded with DNA-coated gold particles (1 μm) and 1,100 psi rupture disc at a stopping-screen-to-target distance of 6 cm, using the Bio-Rad PDS1000/He system. Cells were cultivated for 16–38 h before being harvested and observed. The subcellular localization was determined using an Olympus BX-51 epifluorescence microscope equipped with an Olympus DP-71 digital camera and a combination of YFP and CFP filters. The pattern of localization presented in this work is representative of circa 50 observed cells. The nuclear localizations of the different fusion proteins were confirmed by co-transformation experiments using a nuclear-CFP marker^34^. Such plasmid transformations were performed using 400 ng of each plasmid or 100 ng for BiFC assays.

### Agrobacterium VIGS and qPCR

The THAS1, THAS2, THAS3 and THAS4-HYS silencing fragments were amplified with primers (Supplementary Table 6) and the resulting fragments were cloned into the pTRV2u vector as described^61^. Since THAS4 and HYS are ∼91% identical it was not possible to design silencing fragments to avoid cross-silencing. Therefore, a common silencing fragment for both of the two genes was designed. The resulting pTRV2u constructs were used to silence the different THASs and HYS in *C. roseus* seedlings essentially as described before^35^. Leaves from the first two pairs to emerge following inoculation were harvested from eight plants transformed with the empty pTRV2u and pTRV2u carrying the silencing fragment. The collected leaves were frozen in liquid nitrogen, powdered using a pre-chilled mortar and pestle, and subjected to LC-MS and qRT–PCR analysis. The heteroyohimbine content of silenced leaves was determined by LC-MS. Leaf powder was weighed (10–20 mg), extracted with methanol (2 ml) and vortexed for 1 min. After a 10-min centrifugation step at 17,000*g*, an aliquot of the supernatant (20 μl) was diluted to 200 μl with methanol, filtered through 0.2-μm PTFE filters and analysed on Waters Xevo TQ-MS. The chromatographic separation and MS measurements were carried out as described above (method 2). To more comprehensively assess the global effect of silencing the HYS genes by VIGS on *C. roseus* metabolism, an untargeted metabolomics analysis by LC-MS was performed as previously reported.^24^ However, aside from the changes in the heteroyohimbines reported in Supplementary Fig. 16, no substantial differences in metabolic profiles were noted using this approach.

Gene silencing was confirmed by qRT–PCR. qRT–PCR was also used to check the expression of the other HYS genes to ensure that no cross-silencing occurred. RNA extraction was performed using the RNeasy Plant Mini Kit (Qiagen). RNA (1 μg) was used to synthesize cDNA in 20-μl reactions using the iScript cDNA Synthesis Kit (Bio-Rad). The cDNA served as template for quantitative PCR performed using the CFX96 Real Time PCR Detection System (Bio-Rad) using the SSO Advanced SYBR Green Supermix (Bio-Rad). Each reaction was performed in a total reaction volume of 20 μl containing an equal amount of cDNA, 0.25 mM forward and reverse primers and 1 × Sso Advanced SYBR Green Supermix (Bio-Rad). The reaction was initiated by a denaturation step at 95 °C for 10 min followed by 41 cycles at 95 °C for 15 s and 60 °C for 1 min. Melting curves were used to determine the specificity of the amplifications. Relative quantification of gene expression was calculated according to the delta–delta cycle threshold method using the 40 S ribosomal protein S9 (RPS9). All primer pair (Supplementary Table 7) efficiencies were between 98 and 108%, and the individual efficiency values were considered in the calculation of normalized relative expression, which was performed using the Gene Study feature of CFX Manager Software. All biological samples were measured in technical duplicates.

### pH effect on product profile

Strictosidine was deglycosylated using purified SGD for 25 min at room temperature using assay conditions as described above. Strictosidine aglycone was then incubated at a final concentration of 300 μM at pH 5, 6, 7 and 8 in a buffer mix to avoid buffer ingredient effect on activity ((50 mM phosphate buffer, 50 mM citric acid, 50 mM HEPES). Caffeine (50 μM) was used as an internal standard.

At time 0 the enzyme, either THAS1 or HYS (1 μM final concentration), premixed with NADPH (500 μM) was added to the substrate solution. In parallel, a chemical reducing agent, NaBH_4_ (3 mM final concentration), was added to deglycosylated strictosidine as a control reaction. All reactions were carried out in triplicate. An end-point sample (10 μl) was taken for each assay and prepared for UPLC-MS by addition of 10 μl of 100% MeOH to stop the reaction, and then diluted 1:5 with H_2_O, and centrifuged for 10 min at 4,000 r.p.m. UPLC-MS and data collection were performed as described above for heteroyohimbine separation and quantification.

### CD spectra and analysis

Far ultraviolet CD spectra of the wild-type enzymes THAS1 and HYS, as well as the loop mutants of THAS1 and HYS were recorded on a Chirascan Plus spectropolarimeter (Applied Photophysics) at 20 °C in 10 mM potassium phosphate buffer pH 7.0. Samples were analysed from 180 to 260 nm using a 0.5-nm step at a speed of 1 s per step. Four replicate measurements were performed on each sample and baseline correction was applied to all data. Spectra are presented as the CD absorption coefficient calculated on a mean residue ellipticity basis.

Melting curves of HYS and the HYS loop 2 swap mutant were also acquired by CD. The samples were subjected to temperature ramping at the rate of 1 °C min^−1^ from 20 to 90 °C. Data collection was done from 260 to 201 nm using a 1-nm step and 0.75 s time per point. Data were analysed using the Global 3 software. HYS melting point was measured as 61.0±0.1 °C; enthalpy 351.5±3.6 KJ mol^−1^. HYS loop 2 swap melting point was measured at 62.0±0.1 °C; enthalpy 535.8±4.5 KJ mol^−1^.

### Protein sequence alignments and phylogenetic tree

Protein sequence alignment was generated using ClustalW algorithm with Geneious v.8 (http://www.geneious.com)^62^,^63^. The alignment was edited manually using Seaview V4 (ref. 64) and secondary structure depiction was added using ESPript V3 (http://espript.ibcp.fr)^65^. Phylogenetic analysis was performed using the neighbour-joining^66^ algorithm and Bootstrap analysis with 1,000 replicates.

### Docking of cathenamine in THAS1-NADP^+^ structure

**Figure 6:**
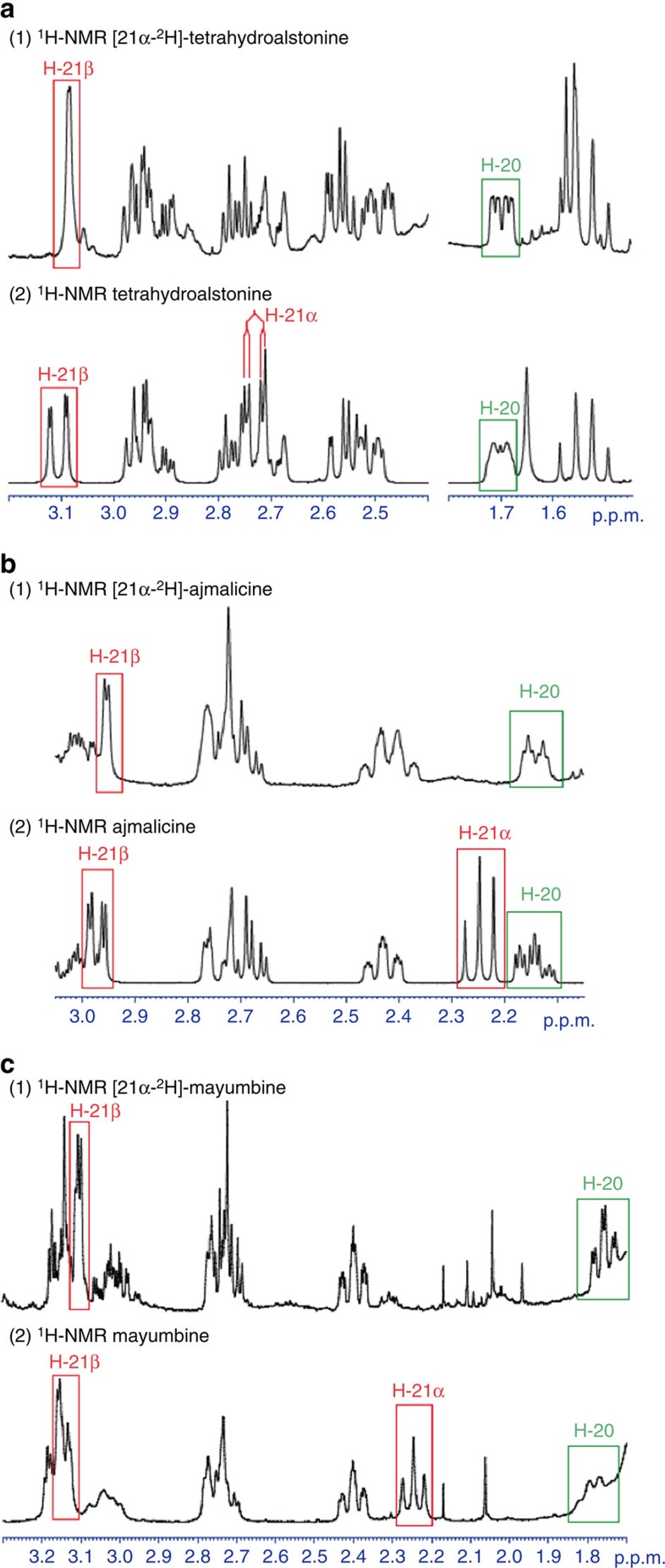
Deuterium labelling of THAS1 and HYS products using pro-*R*-NADPD. Comparison of selected regions of ^1^H-NMR spectra of labelled (**a**) tetrahydroalstonine, (**b**) ajmalicine, (**c**) mayumbine. The spectra indicate that C21 is labelled with deuterium in the pro-*R* position.

**Figure 7:**
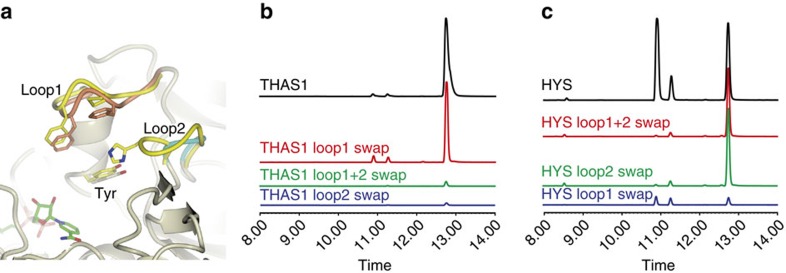
Product profiles of THAS1 and HYS loop swap mutants. (**a**) Shown is the apo THAS1 structure (magnolia) with loops 1 and 2 highlighted in orange and cyan, respectively. For clarity, only the corresponding loops of the HYS apo structure are shown in yellow after superposition. Similarly, only the cofactor from the superposed holo THAS1 structure is shown for reference (green carbons). The side chains of important residues are also shown. (**b**) Representative LC-MS chromatograms of assays with THAS1 mutants in which loop 1, loop 2 or both have been swapped with the corresponding sequences from HYS. (**c**) Representative LC-MS chromatograms of assays with HYS mutants in which loop 1, loop 2 or both have been swapped with the corresponding sequences from THAS1.

**Figure 8:**
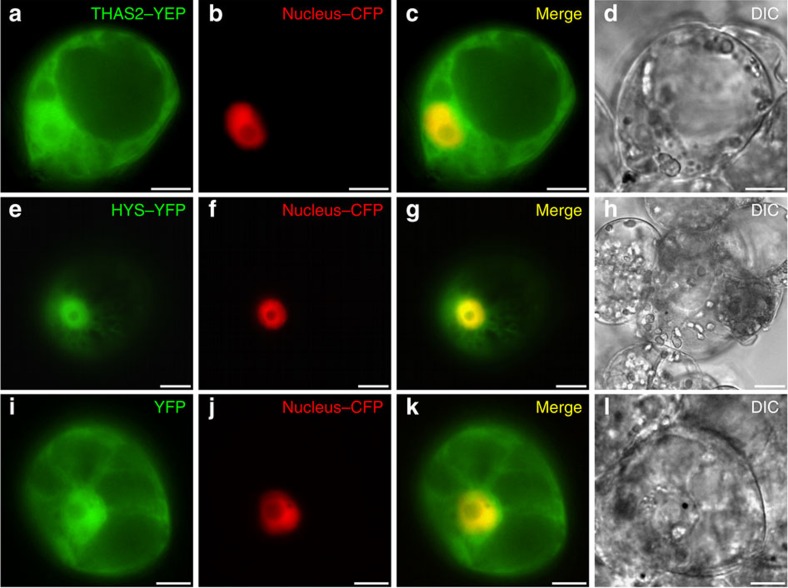
THAS2 displays nucleocytosolic localization while HYS is preferentially targeted to the nucleus. *C. roseus* cells were transiently co-transformed with plasmids expressing either THAS2–YFP (**a**), HYS–YFP (**e**) or YFP (**i**) and the plasmid encoding the nuclear-CFP marker(**b**,**f**,**j**). Co-localization of the fluorescence signals appears in yellow when merging the two individual (green/red) false colour images (**c**,**g**,**k**). Cell morphology is observed with differential interference contrast (DIC) (**d**,**h**,**l**). Scale bars, 10 μm.

**Figure 9:**
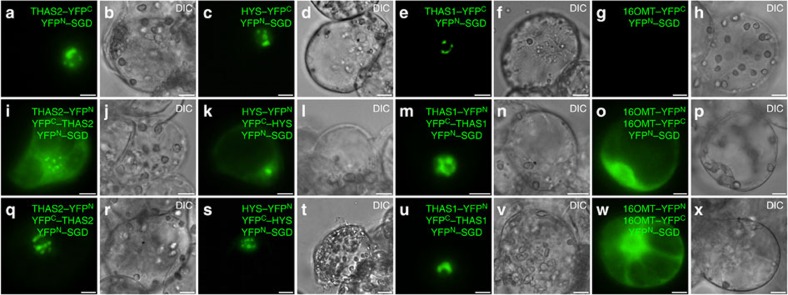
THAS2 and HYS interact with SGD in the nucleus. THAS2/SGD (**a**,**i**,**q**) and HYS/SGD (**c**,**k**,**s**) interactions were analysed by BiFC in *C. roseus* cells transiently transformed by distinct combinations of plasmids encoding fusions with the two split YFP fragments, as indicated on each fluorescence picture. THAS1/SGD (**e**,**m**,**u**) and 16OMT/SGD (**g**,**o**,**w**) interactions were studied to evaluate the specificity of THAS2/SGD and HYS/SGD interactions. Single BiFC assays showing interactions with SGD (upper row) and double BiFC assays highlighting both interactions with SGD and THAS2, HYS, THAS1, 16OMT self-interactions were conducted and observed 16 h (middle row) and 36 h (lower row) post-transformation. Cell morphology is observed with differential interference contrast (DIC) (**b**,**d**,**f**,**h**,**j**,**l**,**n**,**p**,**r**,**t**,**v**,**x**). Scale bars, 10 μm.

Cathenamine was docked into the THAS1-NADP^+^ crystal structure using Autodock 4.2 (ref. 67). The ligand (cathenamine) was prepared with two torsions at the C16, the rest of the molecule being rigid and the receptor consisted of the desolvated high-resolution crystal structure. The search space was defined by a 40 × 40 × 40 Å box with a 0.375-Å grid spacing, centred between the nicotinamide ring and the side chain of Tyr56, and encompassed the entire active site cavity. Searches were performed using the Lamarckian Genetic Algorithm, consisting of 100 runs with a population size of 150 and 2,500,000 energy evaluations. A total of 27,000 generations were analysed and clustered with an RMS tolerance of 2 Å per cluster. This resulted in just two distinct clusters, which constituted 98% and 2% of the resultant poses, respectively. The latter cluster placed the indole moiety such that the nitrogen atom was closest to the cofactor. Thus, this cluster was eliminated since it was inconsistent with the results of the deuterium labelling experiments (Fig. 6). The poses contained within the major cluster were all deemed to be ‘productive' since they placed the indole moiety of cathenamine towards the entrance of the active site and the C20 and C21 3.3 Å above the nicotinamide C4 atom. The top ranked pose (with an estimated free energy of binding=−8.76 kcal mol^−1^), as selected by the software, is used in the structures illustrated here (Fig. 4b).

### Data availability

The atomic coordinates and structure factors of the five X-ray structures described in this manuscript have been deposited in the Protein Data Bank (http://www.pdb.org/), with accession codes 5FI3, 5FI5, 5H81, 5H82 and 5H83. Accession numbers: THAS1 (AKF02528.1); THAS2 (KU865323); THAS3 (KU865322); THAS4 (KU865324); HYS (KU865325); Cro_017994 (KU865326); Cro_011702 (KU865327); Cro_030442 (KU865328); Cro_006840 (KU865329); Cro_022770 (KU865330); Cro_033537 (KU865331); Cro_027234 (AHK60846); Cro_033062 (KU865332); Tabersonine-3-reductase (AKM12281). Data supporting the findings of this study are available within the article and its Supplementary Information files and from the corresponding author upon reasonable request.

## Additional information

**How to cite this article:** Stavrinides, A. *et al*. Structural investigation of heteroyohimbine alkaloid synthesis reveals active site elements that control stereoselectivity. *Nat. Commun.* 7:12116 doi: 10.1038/ncomms12116 (2016).

## Supplementary Material

Supplementary Information Supplementary Figures 1-18, Supplementary Tables 1-8 and Supplementary References.
