# Supplementary material for: Structural investigation of heteroyohimbine alkaloid synthesis reveals active site elements that control stereoselectivity
Source: Nat Commun. 2016 Jul 15;7:12116. doi: 10.1038/ncomms12116 (PMC4947188; doi:10.1038/ncomms12116)
Supplement: Supplementary Information — Supplementary Figures 1-18, Supplementary Tables 1-8 and Supplementary References. [file ncomms12116-s1.pdf]

Supplementary Figures

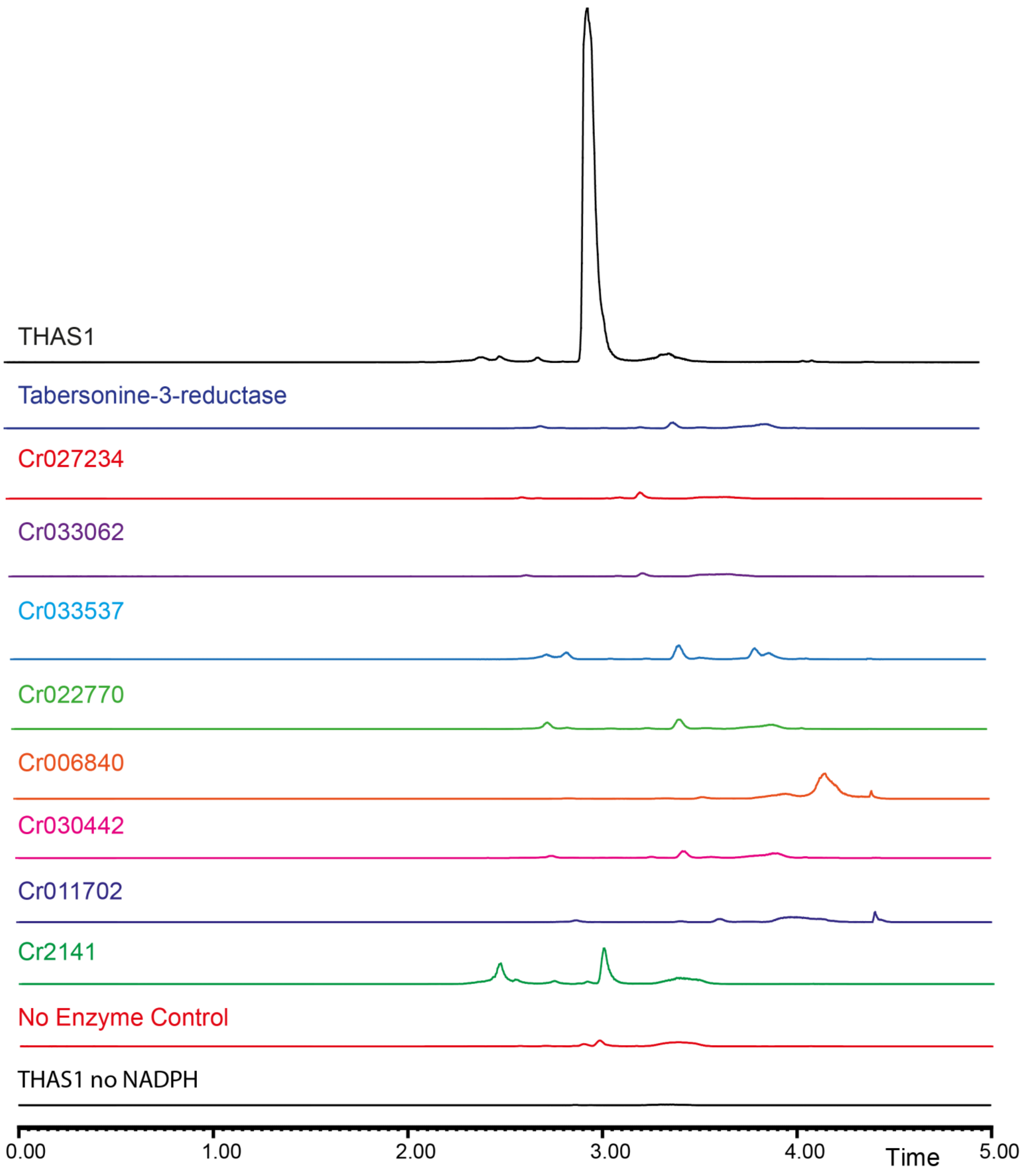

Supplemental Figure 1. LC-MS chromatograms of inactive candidates and selected negative controls. All MDR homologues that were assayed in this study but failed to show significant turnover with strictosidine aglycone are shown in this figure. The function of these enzymes remains unidentified, except for tabersonine-3-reductase, which was recently reported to act in vinblastine biosynthesis (AKM12281.1).[2]

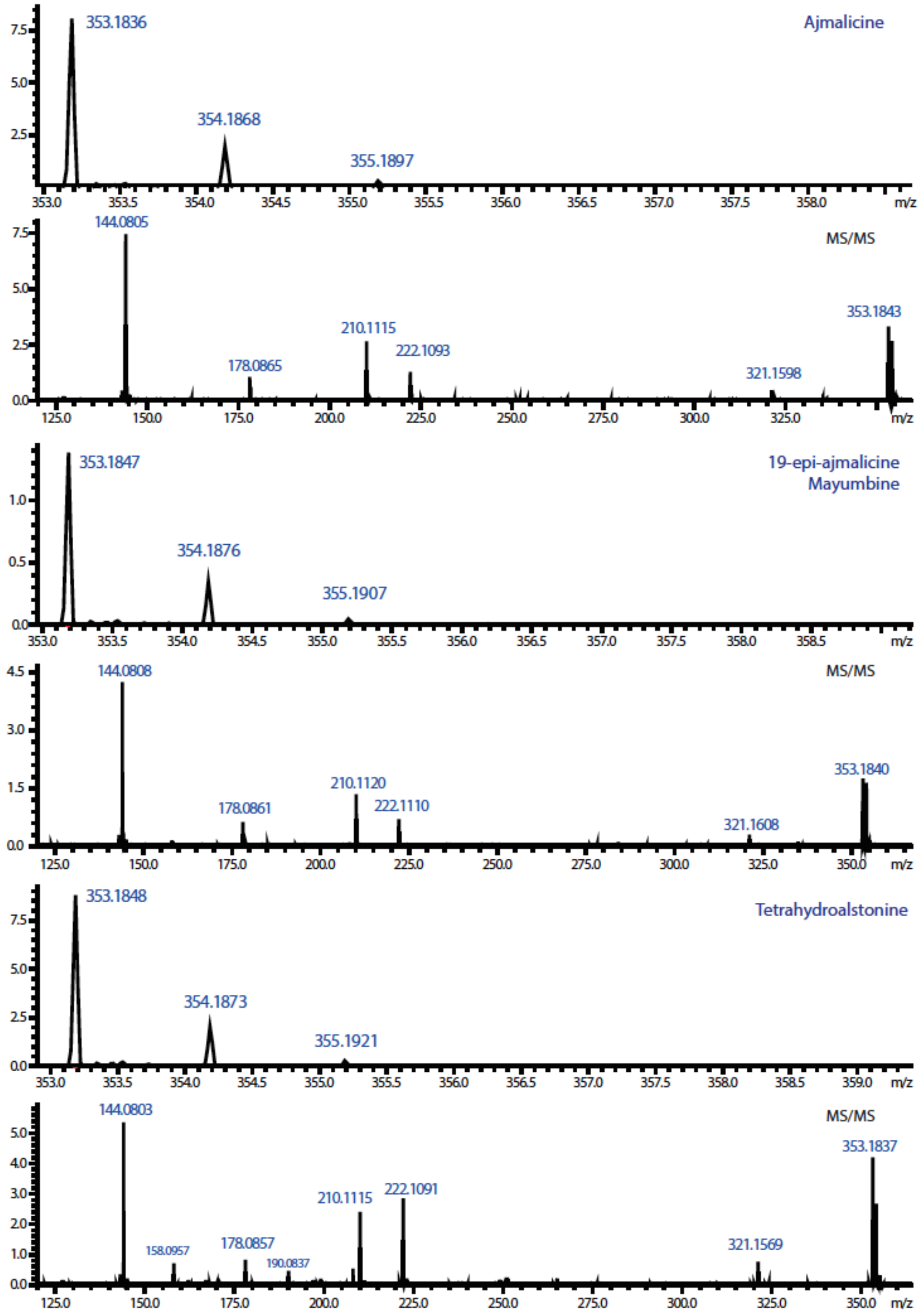

Supplementary Figure 2. High resolution mass spectrometry of HYS enzymatic products. The top panel for each compound shows the high resolution mass; the bottom panel shows the MS/MS spectrum.

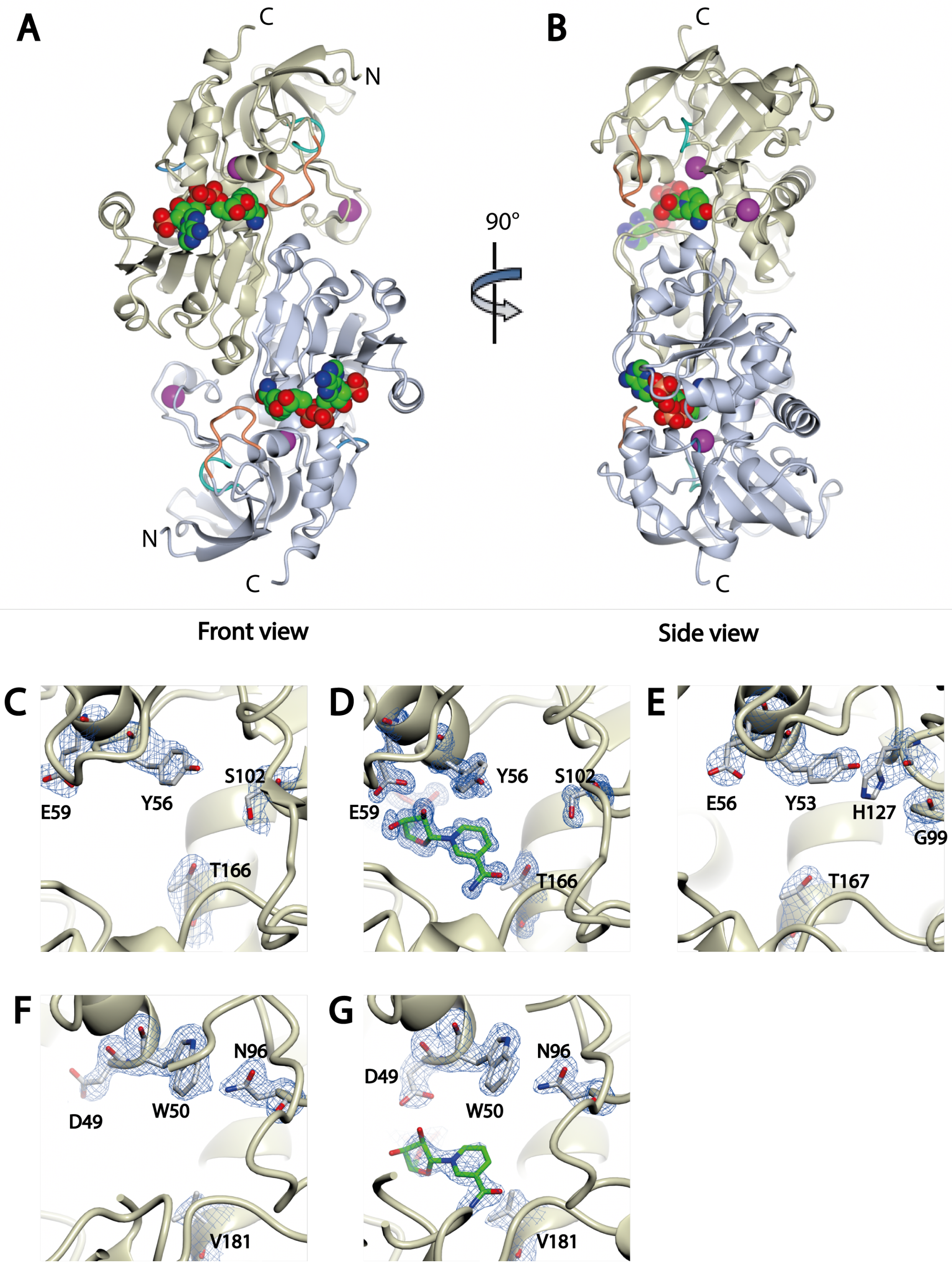
A
B
C
N
90°
Front view
Side view
C
E59
Y56
S102
T166
D
E
E56
Y53
H127
G99
T167
F
D49
W50
N96
V181
G

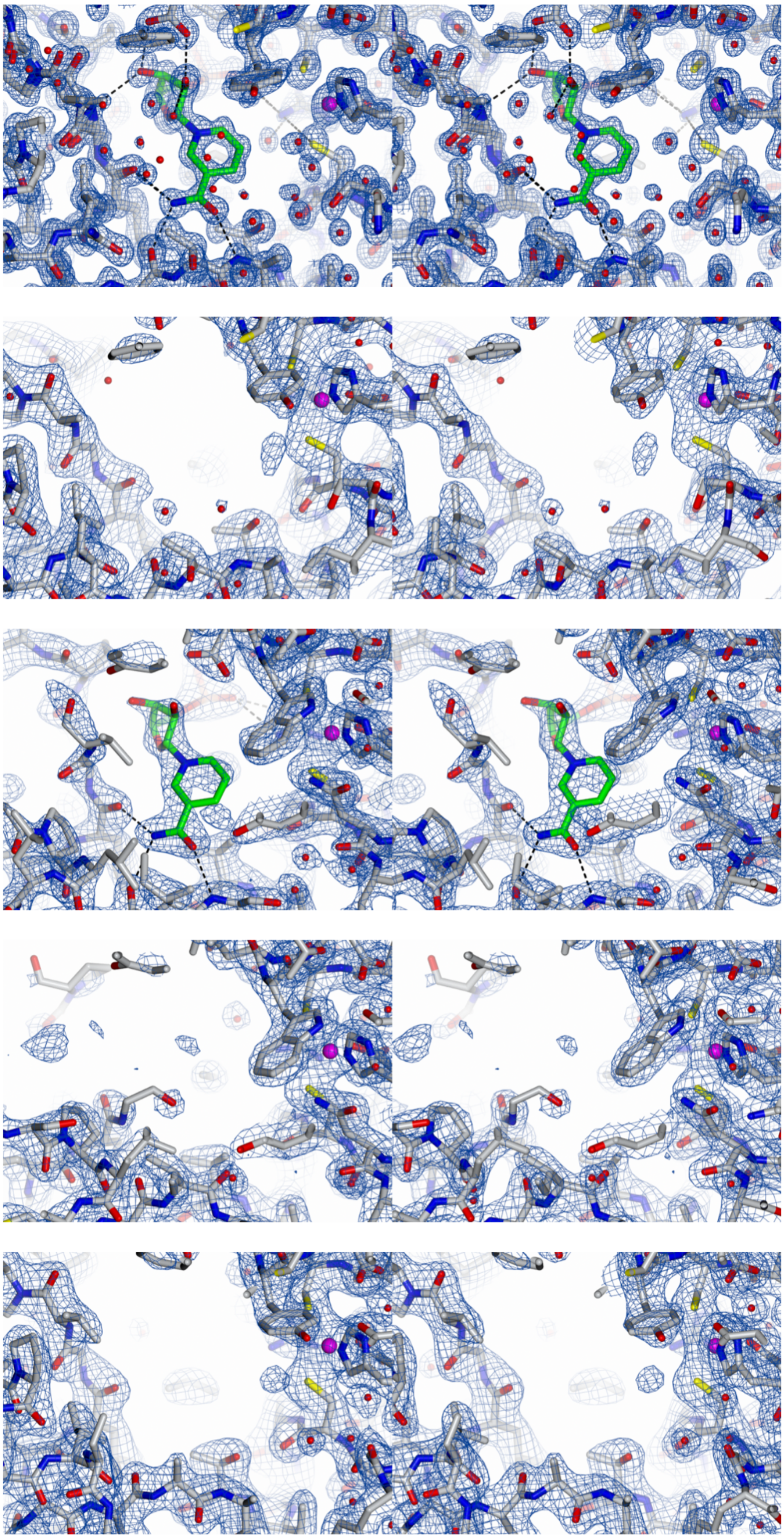

H THAS1 holo (1.05 Å)

I THAS1 apo (2.25 Å)

J THAS2 holo (2.10 Å)

K THAS2 apo (2.05 Å)

L HYS apo (2.15 Å)

Supplementary Figure 3. Overview of THAS1 holo structure. The two subunits of the biological dimer are displayed in cartoon representation and distinguished by magnolia and gray coloration; A: Front view; B: Side view; the $NAPD^+$ cofactors are shown as van der Waals spheres with green carbons, the zinc ions are indicated magenta spheres; loops 1 and 2 are colored in orange and cyan, respectively; the N- and C-termini are labelled where they are clearly visible. C: SA-omit map of THAS1 apo structure; D: SA-omit map of THAS1 holo structure; E: SA-omit map of HYS crystal structure; F: SA-omit map of THAS2 apo structure; G: SA-omit map of THAS2 holo structure. Amino acids of interest are shown in gray carbons; H-L: Stereoviews showing final coordinates and corresponding $2mF_{obs} - dF_{calc}$ electron density maps contoured at ~1.0 σ for all five structures described herein (resolutions given in parentheses). Equivalent views of the active site region are shown in each case.

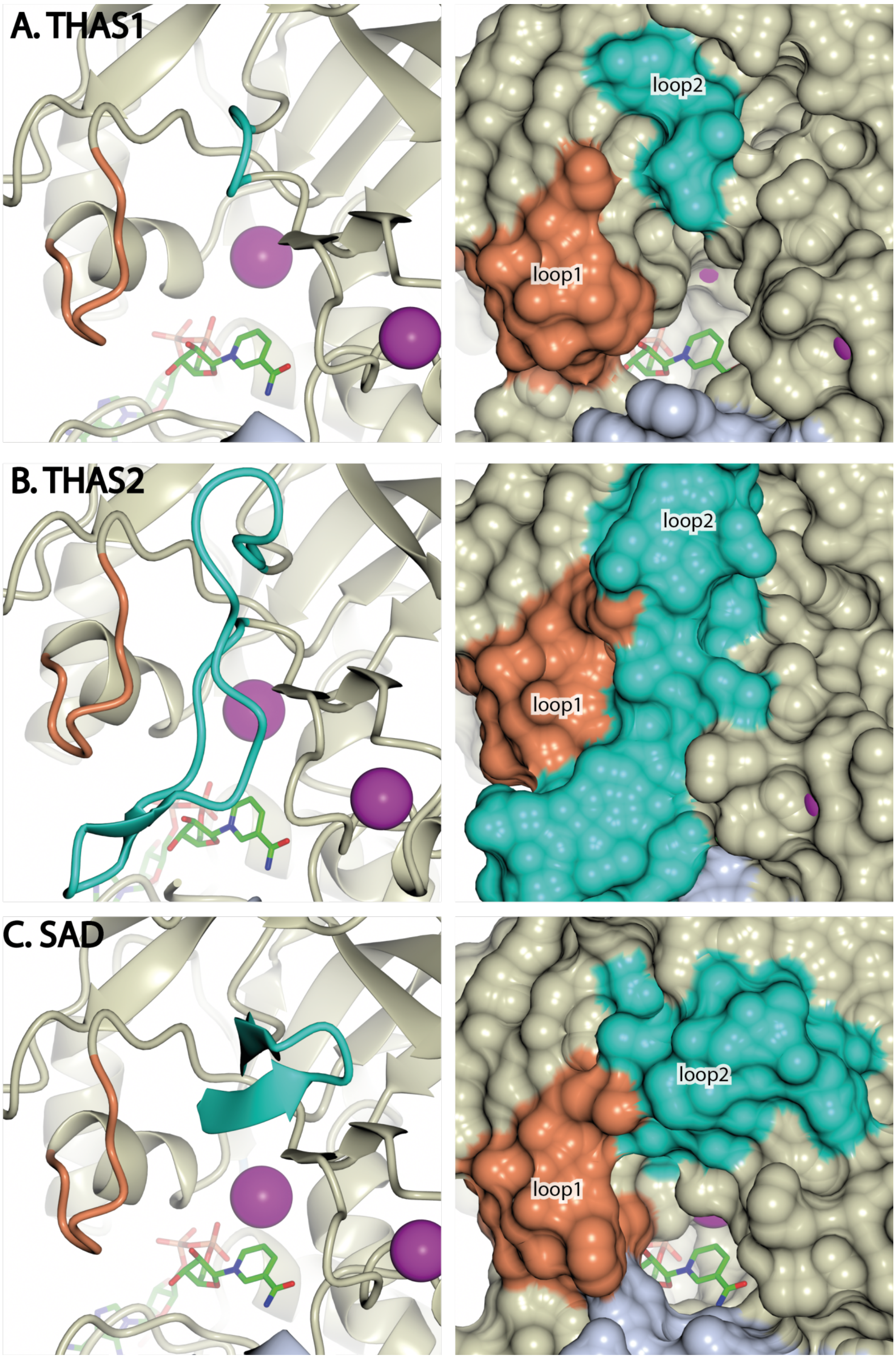

Supplementary Figure 4. Comparison of active site cavities. Protein structures are depicted in cartoon (left panels) and space filling (right panels) representations. The $NADP^+$ cofactor is shown with green carbons; loop 1 is in orange and loop 2 is in cyan. Zinc ions are displayed as magenta spheres. Note the larger cavity in THAS1 relative to SAD and that the proximal zinc is significantly further away from the cofactor in THAS1 (5.2 vs. 3.3 Å). Access to the active site cavity is completely blocked by loop 2 in holo THAS2; presumably this must move aside to allow cofactor and substrate access. Loop 2 is disordered in the THAS2 apo structure, which is consistent with it being flexible (Fig. 4C). The view corresponds approximately to the side view of the top subunit in Supplementary Fig. 3B.

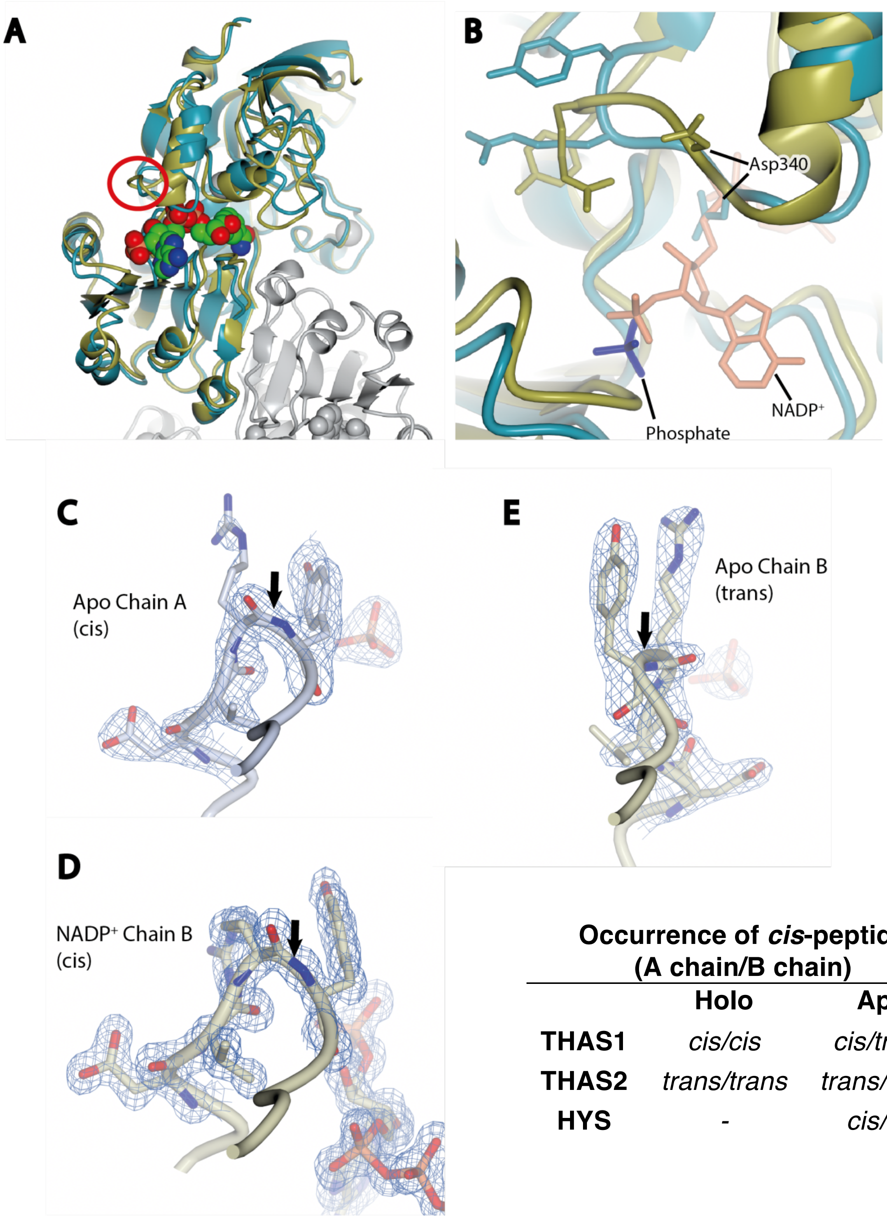

Occurrence of *cis*-peptide (A chain/B chain)

	Holo	Apo
**THAS1**	*cis/cis*	*cis/trans*
**THAS2**	*trans/trans*	*trans/trans*
**HYS**	-	*cis/cis*

Supplementary Figure 5. Non-proline *cis*-peptide bond. A. Superposition of holo (gold) with apo (teal) THAS1 crystal structure based on the full subunit shown. For reference, the second subunit of the holo structure alone is also shown in gray. The lower cofactor binding domains superpose well, while the upper substrate binding domain displays a downward shift upon binding the cofactor. The location of an unusual *cis*-peptide bond that forms between Arg342 and Tyr343 is outlined with a red circle. B. Close-up of the region highlighted in A showing the effect that the conversion from *cis* (gold) to *trans* (teal) has on the conformation of the loop between α-helix 12 and β-strand 18. Most notably, Asp340 flips from an outward facing to an inward facing orientation, thereby placing its side chain in a position that would be incompatible with cofactor (orange) binding due to a steric clash with the pyrophosphate moiety. Also of note is the adventitious binding of a phosphate anion in the apo structure (blue) in a location corresponding to the 2'-phosphate binding site of the cofactor. C. THAS1 apo chain A with *cis*-peptide bond (black arrow); D. THAS1 $NADP^+$ chain B with *cis*-peptide bond (black arrow); E. THAS1 apo chain B with *trans*-peptide bond (black arrow). C, D and E are displayed in the same relative orientation with the structures superimposed on the final $2mF_{obs} - dF_{calc}$ electron density maps contoured at ~1.0 σ. It can only be speculated whether this feature has a role in controlling cofactor binding, or simply reporting the catalytic status of the enzyme. However, the presence of this feature in HYS and its complete absence in the THAS2 structures indicate that its correlation with bound cofactor is not universal.

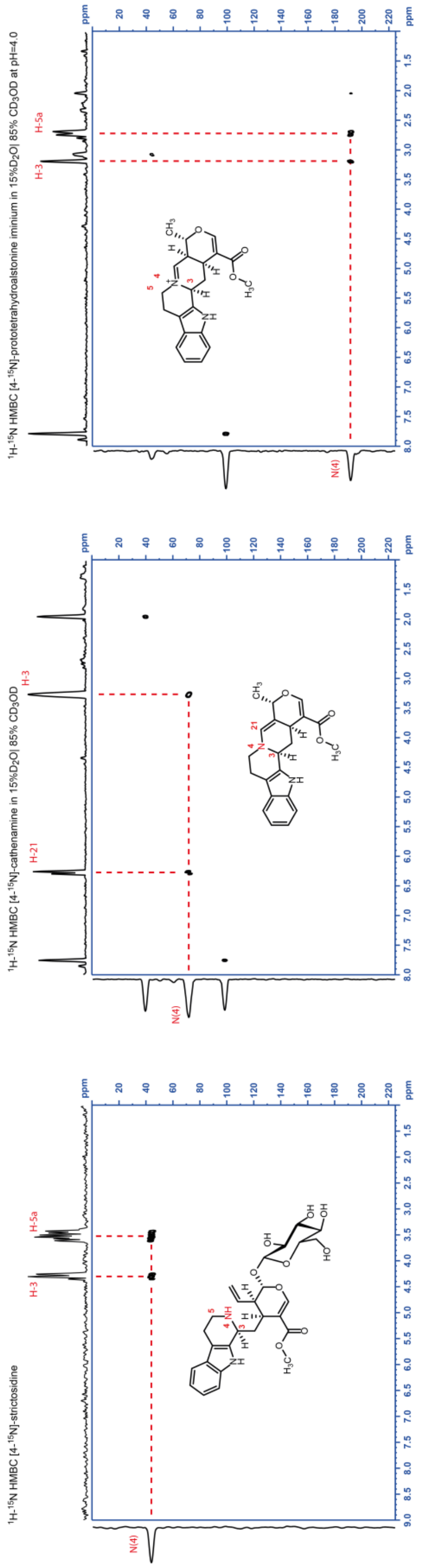

Supplementary Figure 6. ^{1}H,^{15}N-HMBC NMR spectra of ^{15}N labeled products. ^{15}N labeled compounds were synthesized as described in the Methods. ^{15}N resonances for strictosidine, cathenamine and the iminium tautomer of cathenamine that is observed at acidic pH are shown.

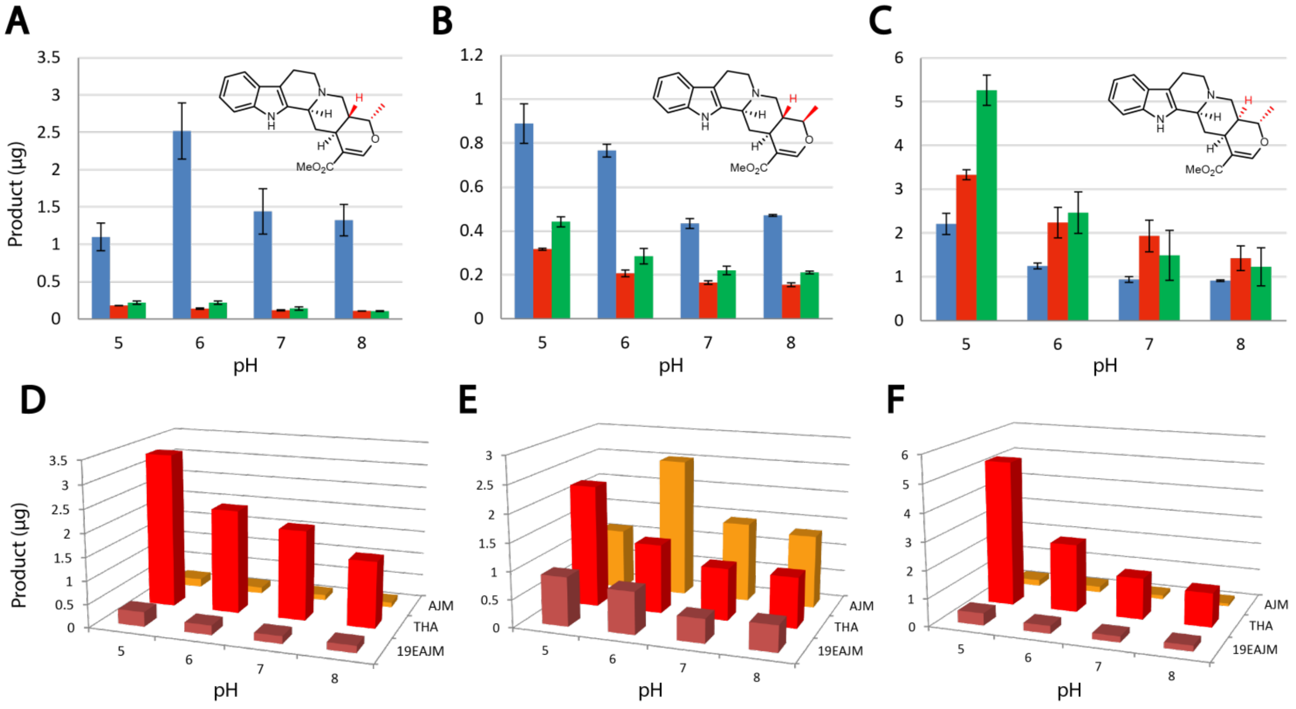

Supplementary Figure 7. Product profile dependence on pH of THAS1, HYS and $NaBH_4$ at pH 5 to 8. Top row: Ajmalicine (A), Mayumbine/19-epiajmalicine (B), and tetrahydroalstonine (C) produced by THAS1 (red), HYS (blue), or $NaBH_4$ (green) per 15.9µg of strictosidine in 10min. Bottom row: 3D graph of products of THAS1 (D), HYS (E), and $NaBH_4$ (F). Error bars are the standard error of the mean of three replicates.

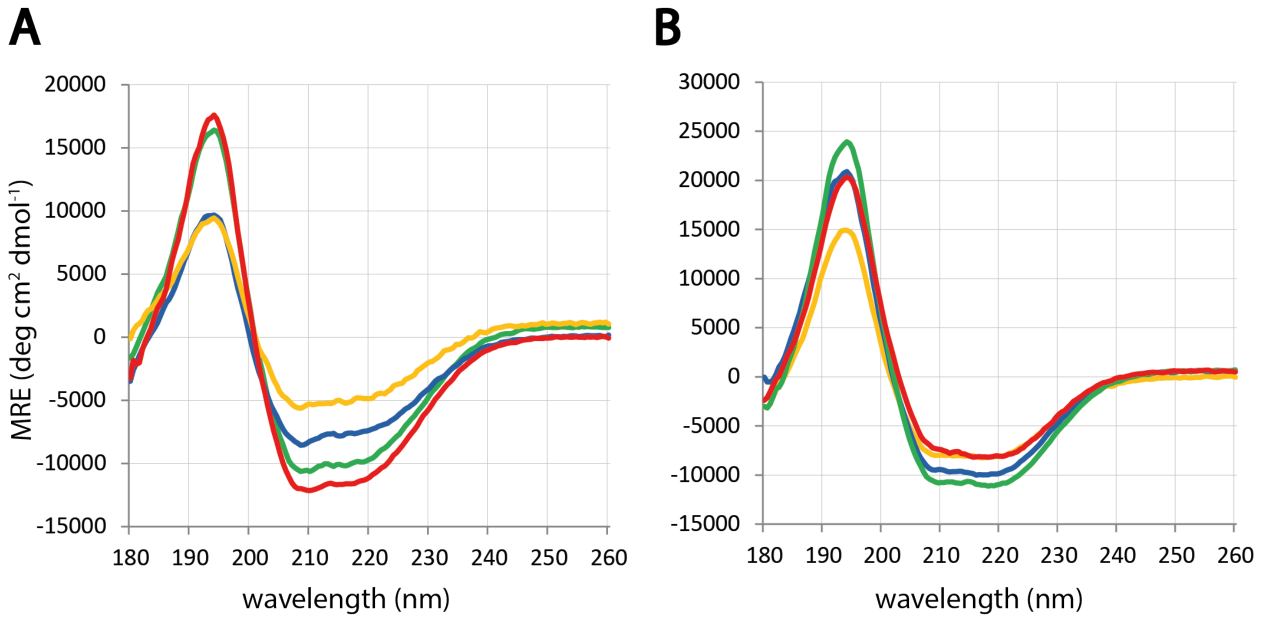

Supplementary Figure 8. Circular dichroism of THAS1, HYS, and the corresponding loop swap mutants. A. WT and loop swap mutants of THAS1. B. WT and loop swap mutants of HYS. Wild-type enzymes are plotted in red, loop 1 swap mutants in green, loop 2 swap mutants are in blue and the double loop 1 and 2 swap mutants are in yellow. The secondary structure of the mutants is not substantially different from that of the wild type proteins.

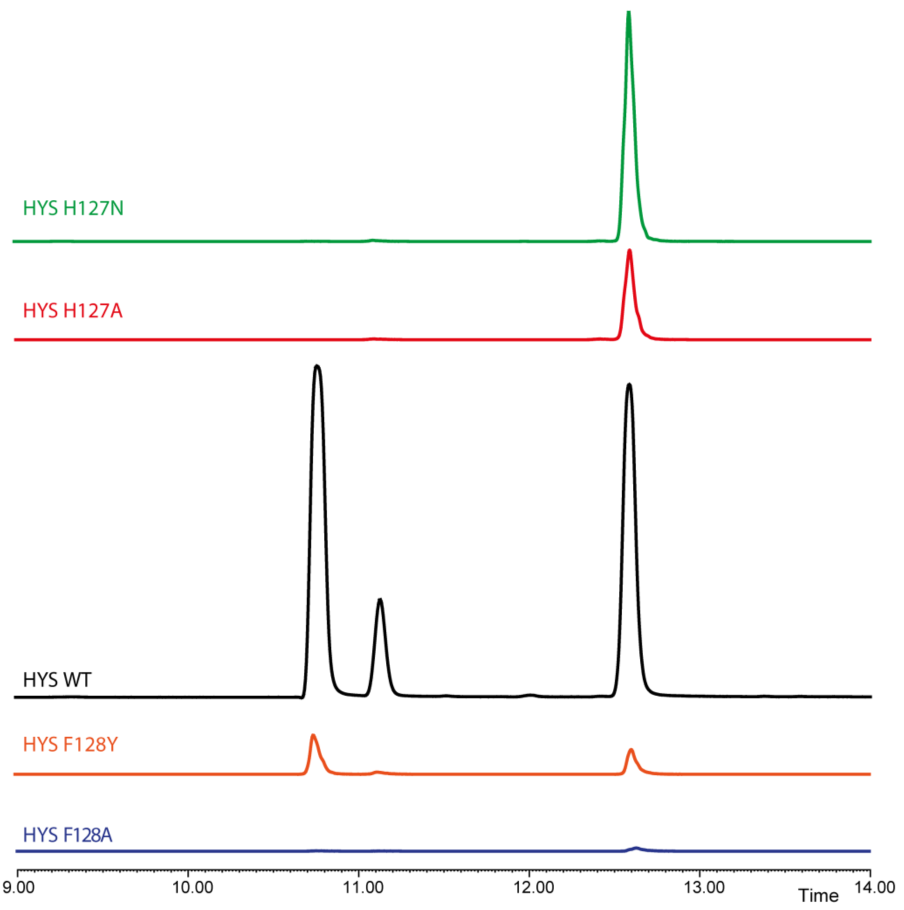

Supplementary Figure 9. Endpoint assays of HYS His127 and F128 mutants. His127, which is present on loop2 in HYS, appears to be responsible for formation of the ajmalicine diastereomer. A second residue on loop 2, F128, was also mutated for comparison, but this mutation simply reduced the activity of the enzyme.

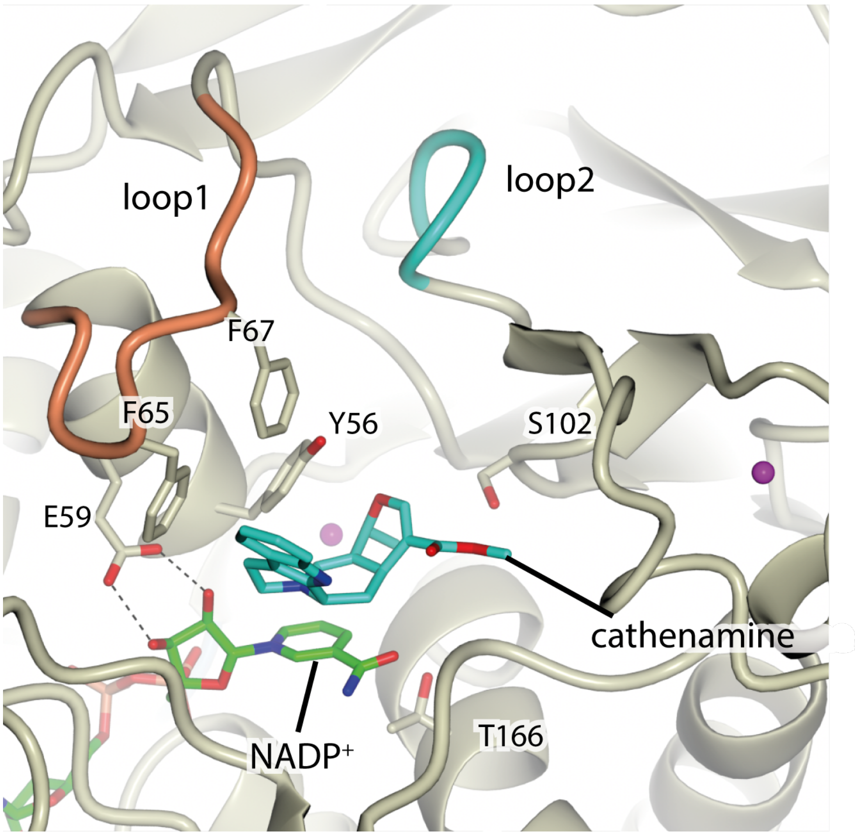

Supplementary Figure 10. Location of mutated residues in active site of THAS1. Loop 1 is indicated by orange, and loop 2 by cyan. The amino acids of interest are drawn in stick representation. Docked cathenamine is also represented in sticks with the carbons in pale blue; the NADP^{+} cofactor is draw in stick representation with green carbons; Zinc atoms are shown as magenta spheres. The side chain of F67 was modelled into the structure for reference.

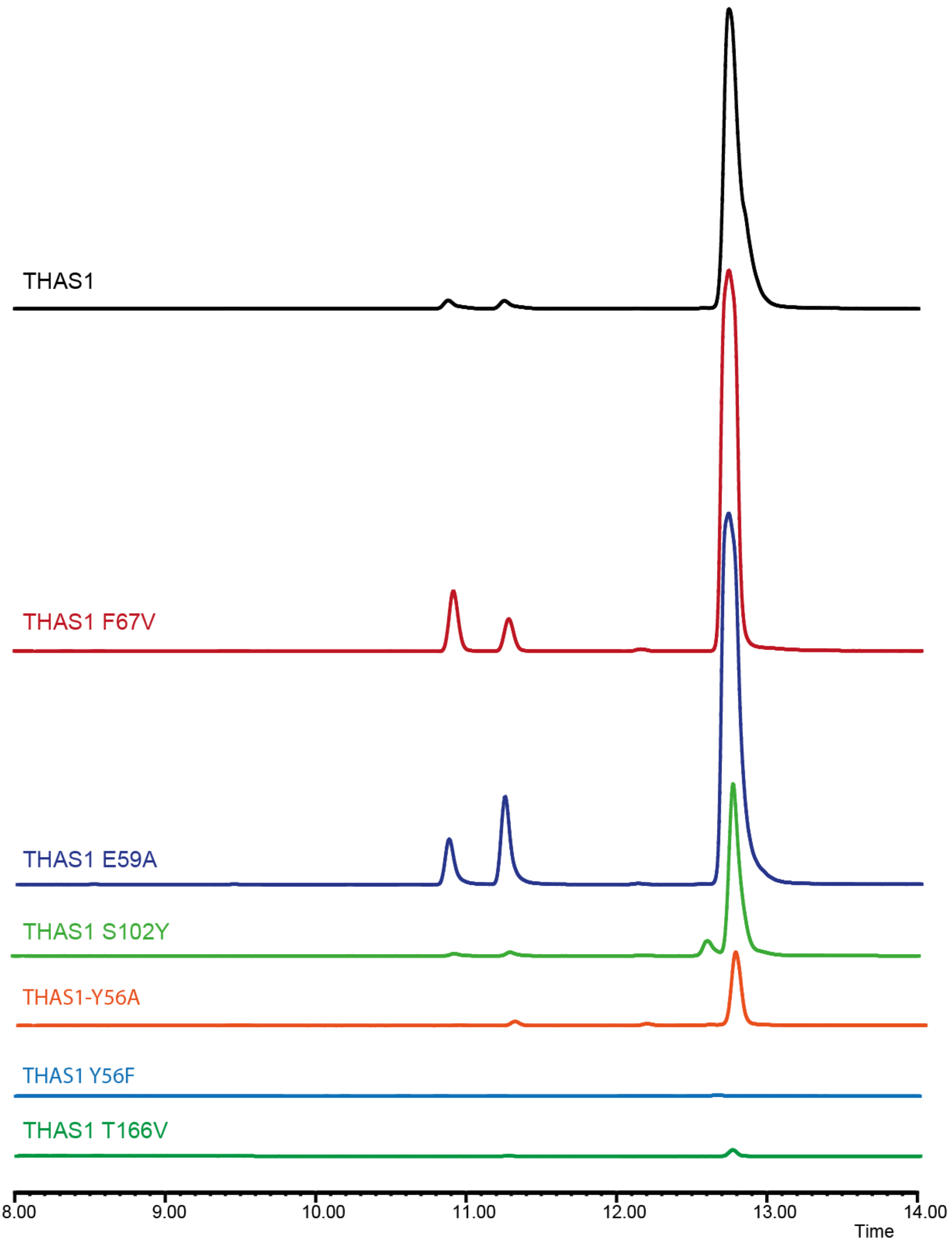

Supplementary Figure 11. Representative chromatograms of selected THAS1 mutants.

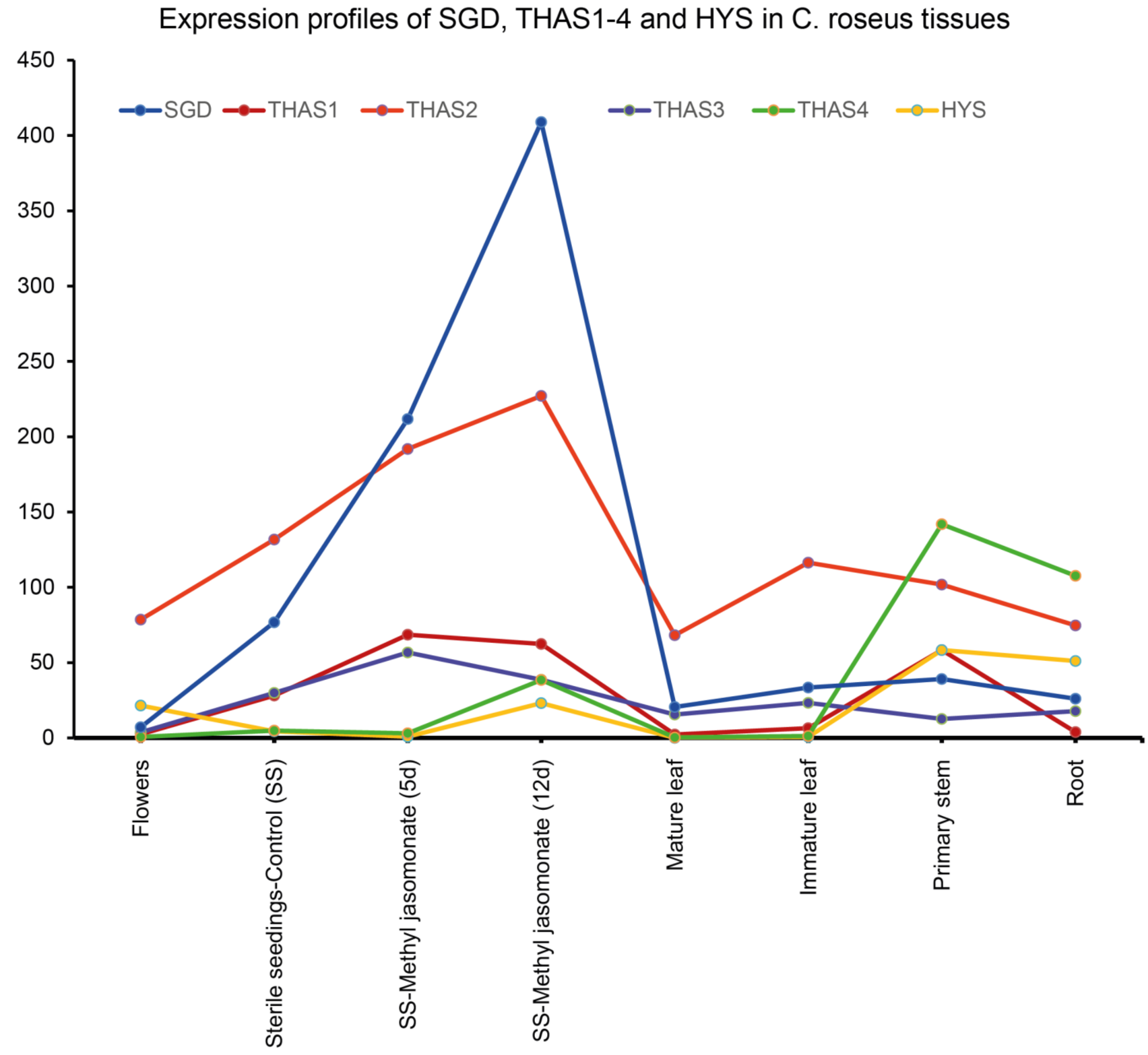

Supplementary Figure 12. Expression profile of SGD, THAS1, THAS2, THAS3, THAS4 and HYS genes according to transcriptomic data (http://medicinalplantgenomics.msu.edu).

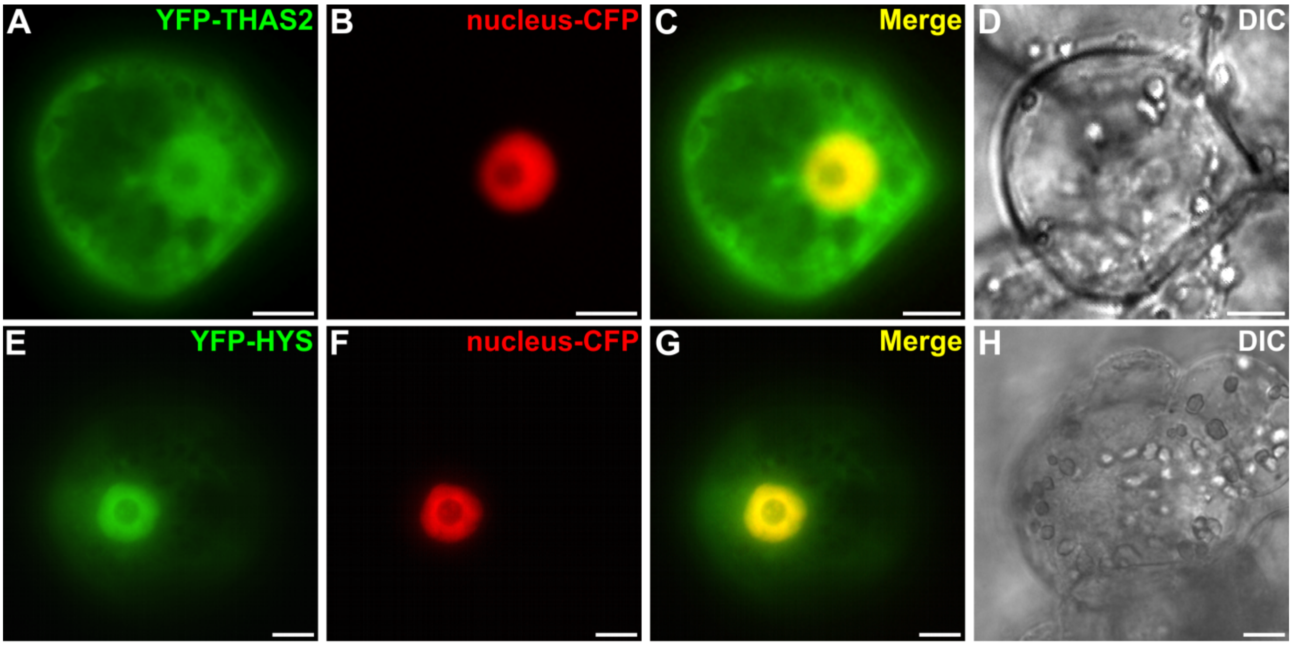

Supplemental Figure 13. THAS2 displays a nucleocytosolic localization while HYS is preferentially targeted to the nucleus. *C. roseus* cells were transiently cotransformed with plasmids expressing either YFP-THAS2 (A) or YFP-HYS (E) and the plasmid encoding the nuclear CFP marker(B, F). Colocalization of the fluorescence signals appears in yellow when merging the two individual (green/red) false color images (C, F). Cell morphology is observed with differential interference contrast (DIC) (D, H). Bars, 10 μm

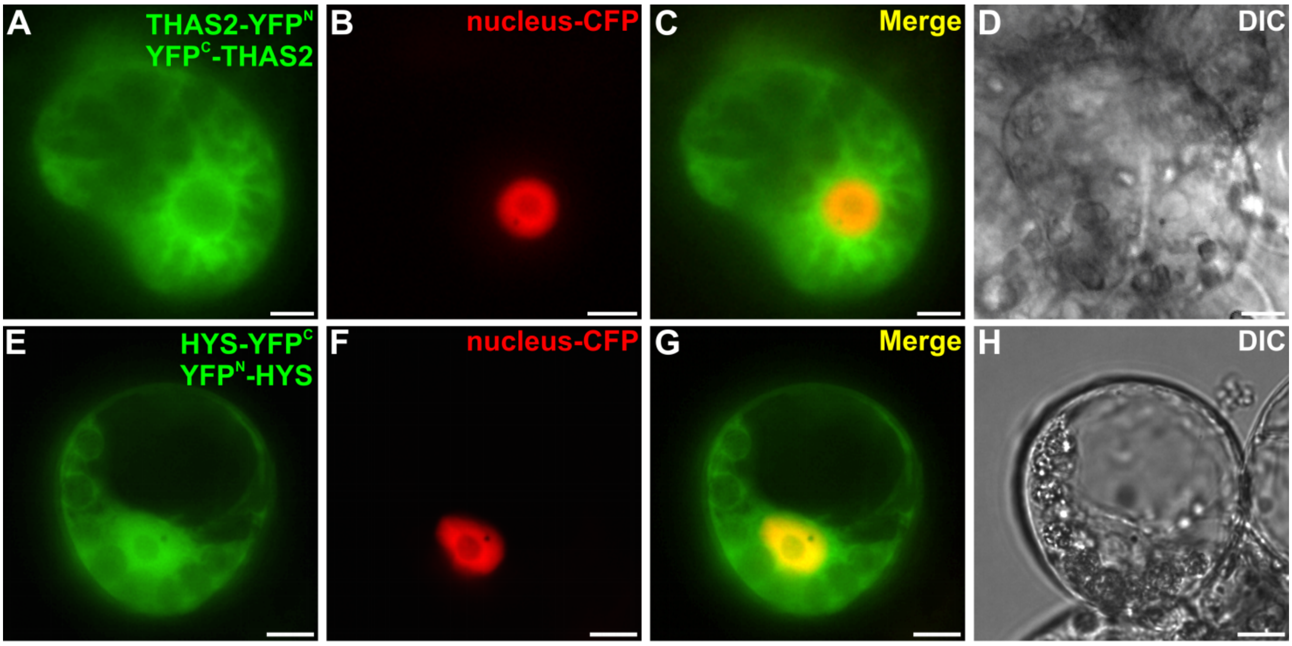

Supplemental Figure 14. THAS2 and HYS self-interactions were studied by BiFC in *C. roseus* cells transiently co-transformed by a combination of plasmids encoding THAS2-YFP^{N} and YFP^{C}-THAS2 (A-D) or HYS-YFP^{N} and YFP^{C}-HYS (E-H) and the plasmids encoding the nuclear CFP marker (B, F). Potential colocalization of the fluorescence signals appears in yellow when merging the two individual (green/red) false color images (C, F). Cell morphology is observed with differential interference contrast (DIC) (D, H). Bars, 10 μm

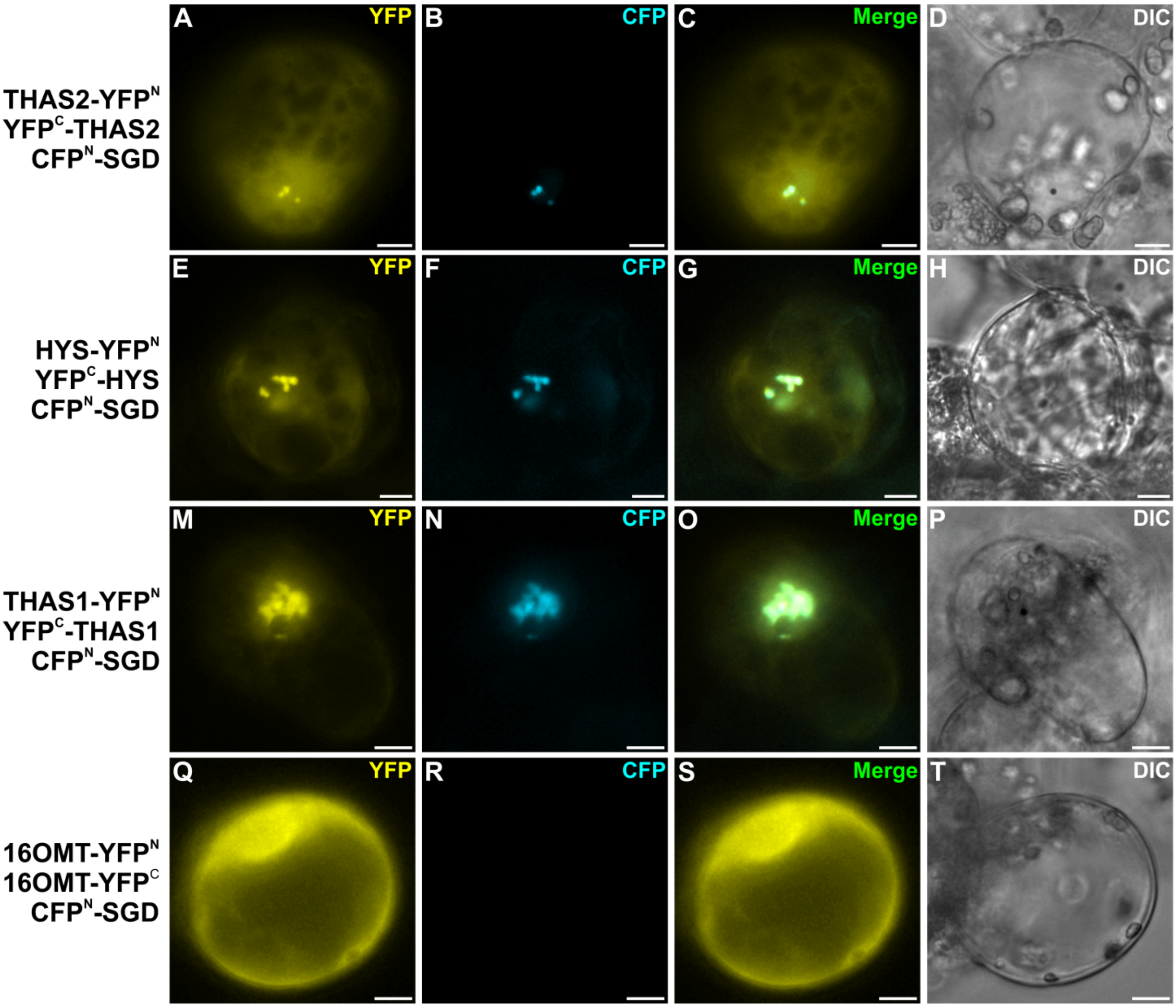

Supplemental Figure 15. THAS2 and HYS self-interactions as well as THAS2/SGD and HYS/SGD interactions were studied by multicolour BiFC. (A-H) *C. roseus* cells were transiently co-transformed by the combinations of plasmids described on the left margin, expressing each hetereoyohimbine synthase fused to both split YFP fragments (YFP^{N} and YFP^{C}) and SGD fused to a split CFP fragment (CFP^{N}-SGD). (M-T) THAS1/SGD and 16OMT/SGD self- and cross-interactions were studied to evaluate the specificity of THAS2/SGD and HYS/ SGD interactions. Association of the two split YFP fragments results in the emission of a yellow fluorescent signal (A, E, M, Q) while association of split CFP^{N} and split YFP^{C} (B, F, N, R) allows the emission of a blue fluorescent signal. THAS2 and HYS self-interactions were observed in both the nucleus and the cytosol and THAS2/SGD and HYS/SGD interactions were only detected in the nucleus. No interactions between THAS2/SGD and HYS/SGD were observed in the cytosol. Cell morphology is observed with differential interference contrast (DIC) (D, H, P, T). Bars, 10 μm

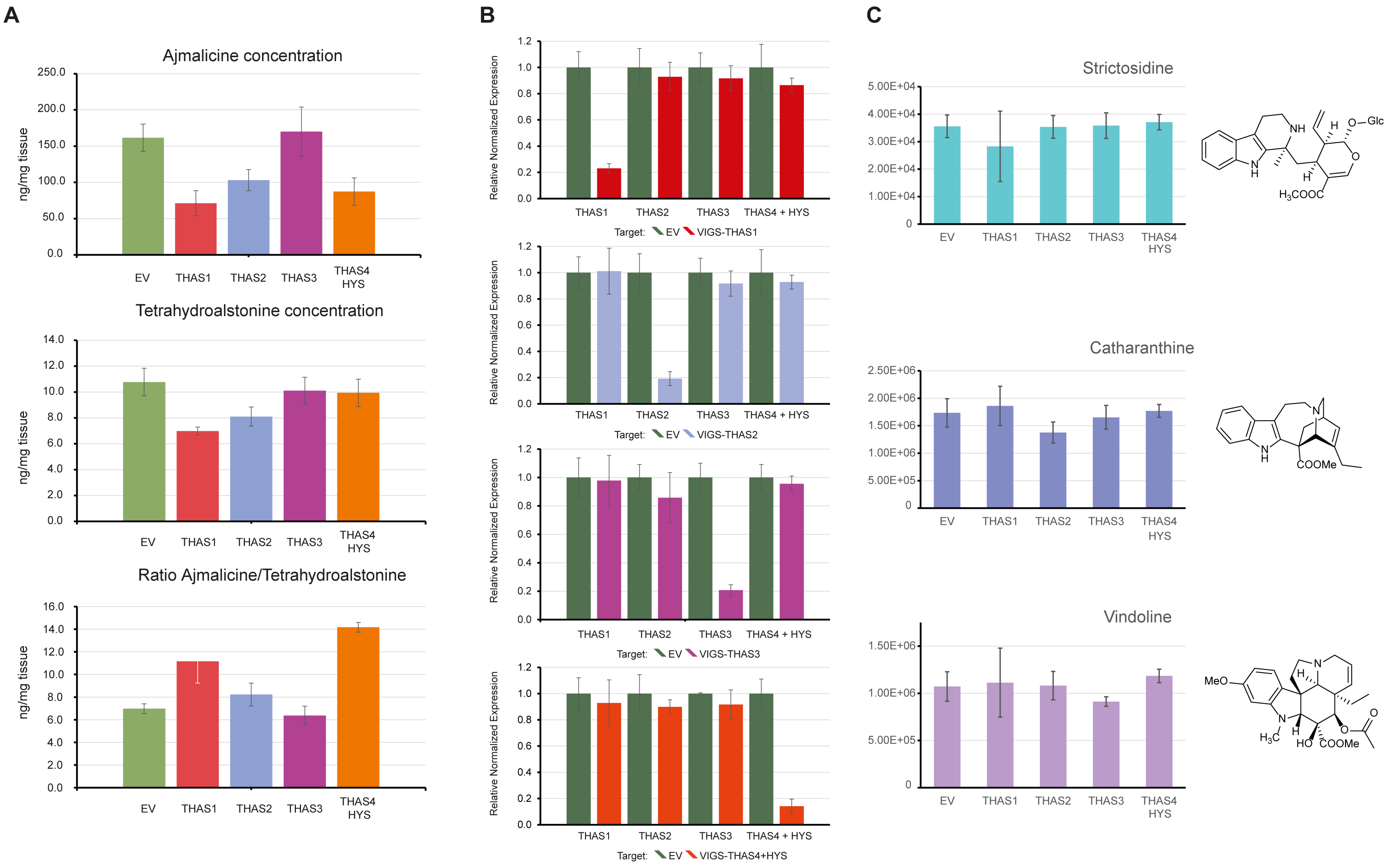

Supplementary Figure 16. VIGS of heteroyohimbine synthases. A. Metabolite analysis of heteroyohimbines in *C. roseus* silenced tissues. Due to high identity of the THAS4 and HYS nucleotide sequence, a common silencing fragment was designed for both genes. B. qRT-PCR data. C. Representative data of non-targeted metabolite analysis. Alkaloids strictosidine, vindoline and catharanthine do not exhibit significant changes in *C. roseus* silenced tissues. Error bars in all cases represent the standard error of eight biological replicates.

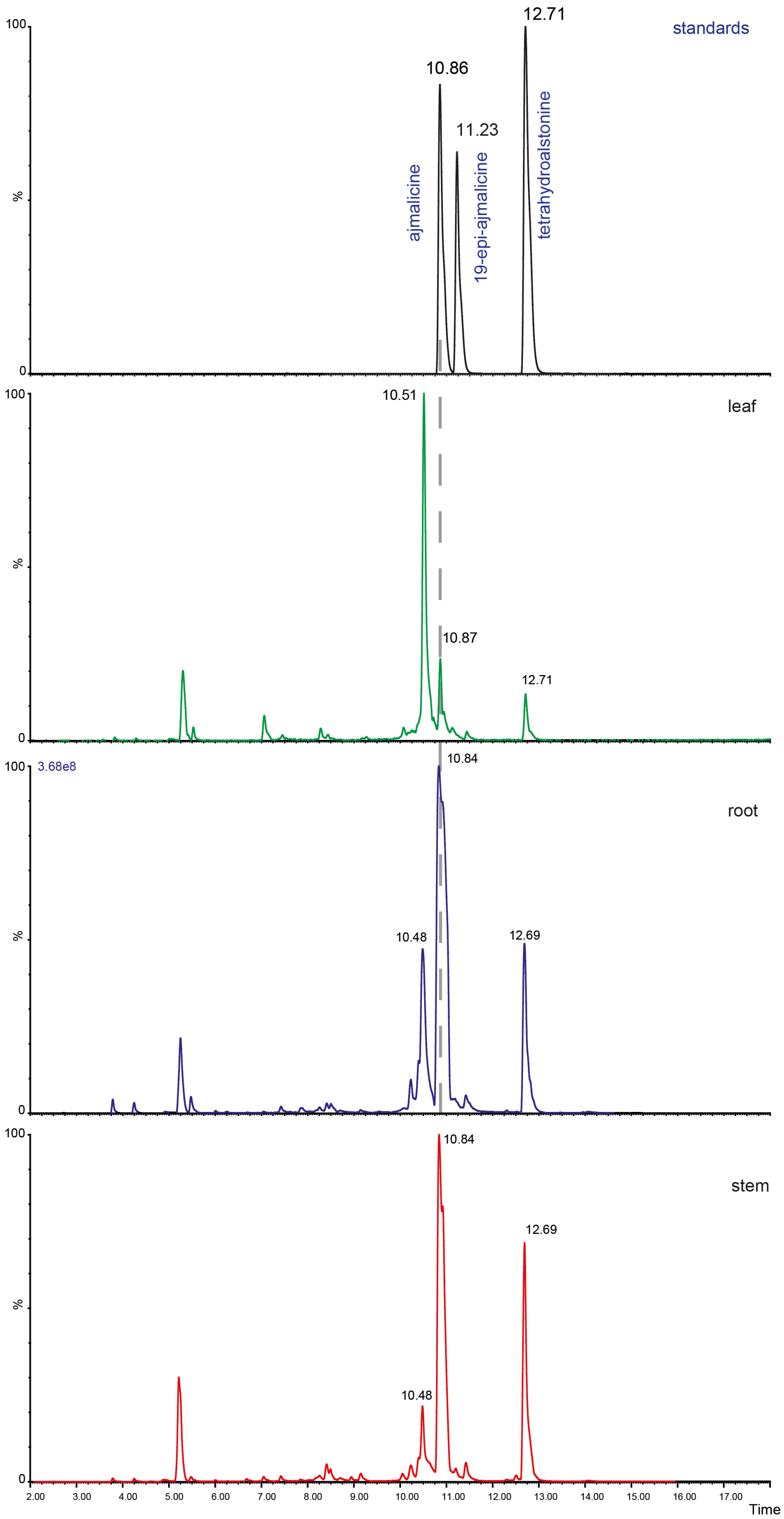

Supplementary Figure 17. Dereplication of heteroyohimbine compounds in different *C. roseus* tissues. Ajmalicine is the most abundant heteroyohimbine in *C. roseus* tissues. Ajmalicine and tetrahydrolastonine are abundant in higher levels in roots and stem. The chromatographic peak with retention time of 10.5 minutes corresponds to an alkaloid that is not in the heteroyohimbine family.

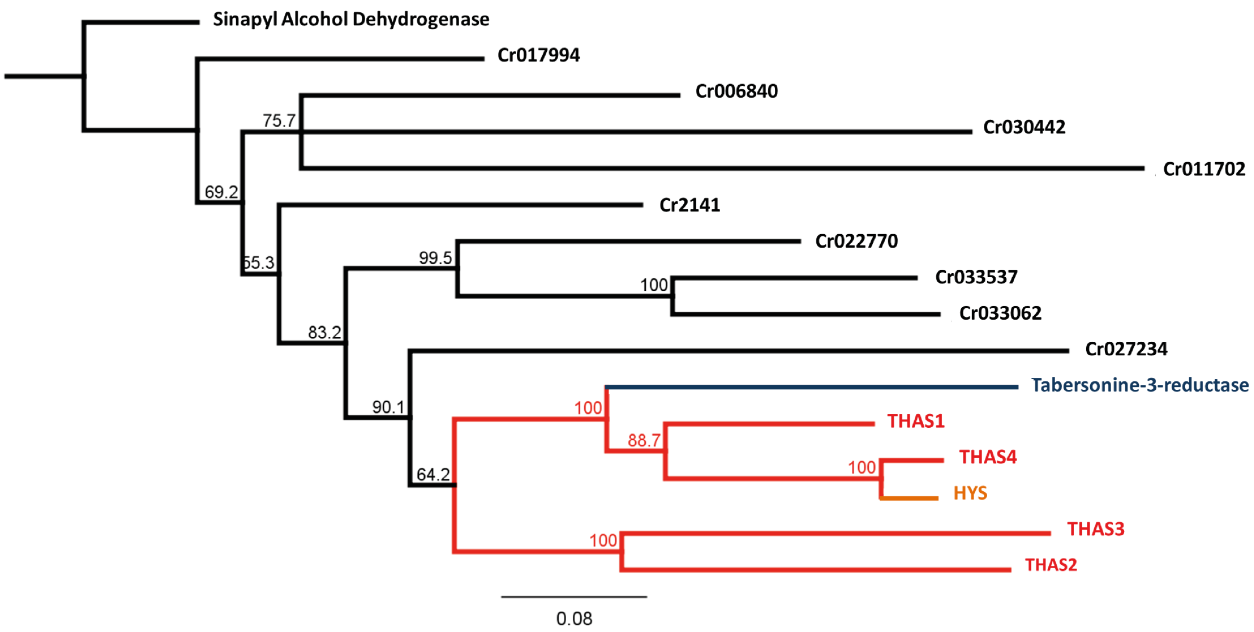

Supplementary Figure 18. Phylogenetic tree illustrating the relationships between the *C. roseus* cloned MDRs and *Populus tremuloides* Sinapyl Alcohol Dehydrogenase (AAK58693.1). Enzymes of particular interest are highlighted: THAS (red); HYS (orange); Tabersonine-3-reductase (blue). Accession numbers: SAD (AAK58693) THAS1 (AKF02528.1); THAS2 (KU865323); THAS3 (KU865322); THAS4 (KU865324); HYS (KU865325); Cro_017994 (KU865326); Cro_011702 (KU865327); Cro_030442 (KU865328); Cro_006840 (KU865329); Cro_022770 (KU865330); Cro_033537 (KU865331); Cro_027234 (AHK60846); Cro_033062 (KU865332); Tabersonine-3-reductase (AKM12281).

Supplementary Tables

Supplementary Table 1. List of candidate MDRs and the primer sequences used to clone these genes into pOPINF vector.

Medium Chain Reductases	Forward primer	Reverse primer
THAS1 Cr024553	AAGTTCTGTTTCAGGGCCCG GCAATGGCTTCAAA	ATGGTCTAGAAAGCTTTAATT TGATTTCAGAGTGTTC
THAS2 Cr021691	AAGTTCTGTTTCAGGGCCCGTCTTC AAAATCAGCAAAACCA	ATGGTCTAGAAAGCTTTAAGCAGATTTCA ATGTGTTTTCTATGTC
THAS3 Cr010119	AAGTTCTGTTTCAGGGCCCGGCAGT TCCATCGGCAGAAACAG	ATGGTCTAGAAAGCTTTAAACAGATCCCA AAGAATTTTCTATATC
THAS4 Cr032583a	AAGTTCTGTTTCAGGGCCCGGCTGC AAAGTCACCTGAAAATG	ATGGTCTAGAAAGCTTTAGAAAGATGGGG ATTTGAGAGTGTTTCCTACG
HYS Cr032583b	AAGTTCTGTTTCAGGGCCCGGCTGC AAAGTCACCTGAAAATG	ATGGTCTAGAAAGCTTTAGAAAGATGGGG ATTTGAGAGTGTTTCCTACG
Cr027234	AAGTTCTGTTTCAGGGCCCGGCTGG AGAAACAAC	ATGGTCTAGAAAGCTTTATTCCTCAAATT TCAATGTATT
Cr033062	AAGTTCTGTTTCAGGGCCCGGCCAG AAAATCACCAGAAGATGAAC	ATGGTCTAGAAAGCTTTACACCTCTGATG GAAGAGTGAGAG
Cr017994	ATGGCTCATAAGAATTGCTTGAATT TTCTT	CTAGATTATGCATTCTTTCTT GAGAGTGTTC
Cr011702	AAGTTCTGTTTCAGGGCCCGGGGAG CTTGGAAGAAGCAG	ATGGTCTAGAAAGCTTTAGTGGTCAACAA GAAGGTTGCTGCC
Cr022770	AAGTTCTGTTTCAGGGCCCGGCTGG GAAATCACCAGAAG	ATGGTCTAGAAAGCTTTAAGACTCCGGTG GAGGAGTTAAAGTG
Cr030442	AAGTTCTGTTTCAGGGCCCGGCTCA AACAACTCCAAACCATAC	ATGGTCTAGAAAGCTTTAAAGATTAGATG ATTTGGAAGCTATATCGATC
Cr006840	AAGTTCTGTTTCAGGGCCCGGCAAA GACACCAGAAACAGAGC	ATGGTCTAGAAAGCTTTATGGACTGGATA ATGAGTTCGCC
Cr033537	AAGTTCTGTTTCAGGGCCCGGCCGG AAAATCAGCAGAAGAAGAA	ATGGTCTAGAAAGCTTTATAACTCTGACG GAGGAGTCAAGGTATT
Cr2141	AAGTTCTGTTTCAGGGCCCGGCCGG AAAATCACCAGAAGAG	ATGGTCTAGAAAGCTTTAAGGAGCTTTCA AGGTCTTTGCAACG
T3R	AAGTTCTGTTTCAGGGCCCGGCTGC AAAGTCAGTGAAGGC	ATGGTCTAGAAAGCTTTAAAAATAGATAG GGTGATTTGAAAGTGTTTCC

Supplementary Table 2. Results of enzyme assay analysis by LCMS of MDRs (*C. roseus*) that were screened for heteroyohimbine synthase activity. The experimental conditions of enzyme assays and the analytical conditions are reported in Methods. The total conversion refers to the yield compared to the starting concentration of strictosidine aglycone. The measured concentrations of the three identified heteroroyohimbines (ajmalicine, mayumbine, tetrahydroasltonine) define the product ratio. The conversion of all enzymes was compared to conversion of THAS1.

MDRs	Total conversion	Product ratio			Conversion compared to THAS1
		Ajmalicine	Mayumbine	Tetrahydroalstonine	
THAS1 Cr024553	18.89%	1.5%	1.77%	96.7%	100.00%
THAS2 Cr021691	19.01%	1.5%	11.87%	86.6%	29.48%
THAS3 Cr010119	23.20%	0.4%	11.88%	87.7%	122.75%
THAS4 Cr032583a	10.74%	0.9%	10.28%	88.9%	56.87%
HYS Cr032583b	21.81%	35.2%	15.09%	49.7%	115.49%
Cr027234	0.07%	0.00%	0.00%	100.0%	0.37%
Cr033062	0.45%	16.7%	4.87%	78.4%	2.36%
Cr011072	0.04%	0.00%	0.00%	100.0%	0.21%
Cr022770	0.04%	0.00%	0.00%	100.0%	0.19%
Cr030442	0.03%	0.00%	0.00%	100.0%	0.15%
Cr006840	0.02%	0.00%	0.00%	100.0%	0.12%
Cr033537	0.15%	0.00%	0.00%	100.0%	0.81%
T3R	0.13%	0.00%	0.00%	100.0%	0.67%
Cr2141	0.01%	0.00%	0.00%	100.0%	0.07%

Supplementary Table 3. X-ray data collection and refinement statistics.

	**THAS1 NADP$^+$** 5FI3	**THAS1 apo** 5FI5	**THAS2 NADP$^+$** 5H81	**THAS2 apo** 5H82	**HYS apo** 5H83
**Data collection**					
Space group	$P2_12_12$	$P3_121$	$P2_12_12_1$	$P2_12_12_1$	I4
Cell dimensions					
a, *b*, *c* (Å)	102.41,112.31,57.11	88.93,88.93,188.12	70.96,83.79,117.16	72.36,88.08,121.39	188.63,188.63,58.95
α, β, γ (°)	90.00,90.00,90.00	90.00,90.00,120.00	90.00,90.00,90.00	90.00,90.00,90.00	90.00,90.00,90.00
Resolution (Å)*	40.04-1.05 (1.08-1.05)	71.27-2.25 (2.31-2.25)	35.48-2.10 (2.15-2.10)	60.69-2.05 (2.10-2.05)	48.32-2.25 (2.31-2.25)
R_{merge}*	0.059 (1.290)	0.123 (1.959)	0.106 (0.905)	0.140 (1.882)	0.140 (1.366)
$I / \sigma I$*	15.4 (1.4)	14.0 (1.4)	16.5 (2.4)	17.0 (1.9)	9.1 (1.1)
Completeness (%)*	99.7 (96.9)	99.0 (97.8)	99.9 (99.8)	99.9 (99.9)	99.7 (97.8)
Redundancy*	8.8 (6.8)	11.2 (11.3)	7.4 (7.6)	18.8 (19.2)	5.1 (5.0)
**Refinement**					
Resolution (Å)*	40.04-1.05 (1.08-1.05)	71.27-2.25 (2.31-2.25)	35.48-2.10 (2.15-2.10)	60.69-2.05 (2.10-2.05)	48.32-2.25 (2.31-2.25)
No. reflections	289489	39037	39326	46829	46849
R_{work} / R_{free}	0.123 / 0.143	0.203 / 0.246	0.186 / 0.232	0.186 / 0.225	0.190 / 0.224
No. atoms					
Protein	5547	5052	5242	5133	5235
Ligand/ion	108	14	100	4	4
Water	878	144	291	320	180
B-factors (Å^2)					
Protein	16.4	60.7	35.8	44.1	54.2
Ligand/ion	13.1	71.5	45.0	39.7	51.9
Water	31.0	50.9	37.1	42.4	42.3
R.m.s. deviations					
Bond lengths (Å)	0.012	0.011	0.011	0.012	0.010
Bond angles (°)	1.64	1.48	1.47	1.49	1.42

*Values in parentheses are for highest-resolution shell.

Supplementary Table 4. Summary of structural superpositions.

**RMSD in Å (number of residues aligned)**	**THAS1 NADP⁺** 5FI3	**THAS1 apo** 5FI5	**THAS2 NADP⁺** 5H81	**THAS2 apo** 5H82	**HYS apo** 5H83	**SAD NADP⁺** 1YQD	**CAD NADP⁺** 2CF6	**CAD apo** 2CF5
**THAS1 NADP⁺** 5FI3	0.40 (344)	1.21 (337)<br>1.54 (661)	1.32 (326)<br>3.40 (588)	1.38 (325)<br>3.11 (591)	1.50 (337)<br>2.28 (660)	0.84 (342)<br>2.48 (655)	1.29 (338)<br>1.72 (663)	1.56 (335)<br>1.87 (650)
**THAS1 apo** 5FI5		0.43 (338)	0.97 (332)<br>3.47 (586)	0.91 (327)<br>3.54 (584)	0.72 (342)<br>1.48 (677)	1.57 (338)<br>2.81 (630)	1.50 (338)<br>2.05 (661)	1.46 (335)<br>1.86 (661)
**THAS2 NADP⁺** 5H81			0.31 (336)	0.53 (333)<br>1.78 (651)	1.22 (333)<br>4.01 (563)	1.42 (326)<br>2.10 (646)	1.49 (330)<br>2.10 (646)	1.59 (332)<br>3.51 (576)
**THAS2 apo** 5H82				0.38 (333)	1.07 (331)<br>4.32 (564)	1.52 (325)<br>2.31 (616)	1.54 (326)<br>3.06 (591)	1.61 (329)<br>3.59 (573)
**HYS apo** 5H83					0.37 (348)	1.74 (340)<br>3.46 (626)	1.63 (339)<br>2.90 (662)	1.61 (341)<br>2.47 (673)
**SAD NADP⁺** 1YQD						0.26 (359)	1.44 (342)<br>2.65 (636)	1.79 (342)<br>3.06 (624)
**CAD NADP⁺** 2CF6							0.00 (352)*	0.86 (351)<br>1.15 (696)
**CAD apo** 2CF5								0.00 (352)*

Pairwise monomer (top row) and dimer (bottom row) superpositions of all protein structures determined herein together with SAD and CAD structures; RMSD values were determined by the Secondary Structure Matching algorithm within SUPERPOSE.[1] Within a given structure only A to B chain monomer superpositions are shown (gray shading) and for the CAD structures the subunits within the dimer were related by crystallographic symmetry and therefore identical (*hence the RMSD values are zero). Elsewhere, A to A chain monomer superpositions were made, and the values determined for the closest match were reported for the dimer superpositions. Orange shading indicates holo to apo comparisons for the same protein; blue shading indicates all holo to holo comparisons; green shading indicates all apo to apo comparisons. For brevity the lower resolution structure of SAD with NADP⁺ bound (PDB accession code 1YQX) was not included in the comparisons

Supplementary Table 5. List of primer pairs for mutagenesis of THAS1 and HYS.

Mutant	*Forward primer*	*Reverse primer*
THAS1 mutants		
Y56A	GGACTTGCCAAGCTGACAGGGAAATGAGCAAAAACAAATTTGG	CCAAATTTGTTTTTGCTCATTTCCCTGTCAGCTTGGCAAGTCC
Y56E	GACAGGGAAATGAGCAAAAACAAATTTGG	CCAAATTTGTTTTTGCTCATTTCCCTGTCTTCTTGGCAAGTCCC
Y56T	GACAGGGAAATGAGCAAAAACAAATTTGG	CCAAATTTGTTTTTGCTCATTTCCCTGTCAGTTTGGCAAGTCCC
Y56S	GACAGGGAAATGAGCAAAAACAAATTTGG	CCAAATTTGTTTTTGCTCATTTCCCTGTCACTTTGGCAAGTCCC
Y56F	GACAGGGAAATGAGCAAAAACAAATTTGG	CCAAATTTGTTTTTGCTCATTTCCCTGTCAAATTGGCAAGTCCC
E59A	GGACTTGCCAATATGACAGGGCAATGAGCAAAAACAAATTTGG	CCAAATTTGTTTTTGCTCATTGCCCTGTCATATTGGCAAGTCC
S102A	CGGGGACAAAGTGGGCGTAGCAGCCATAATTGAAACTTGTGG	GCTACGCCCACTTTGTCCCCG
S102Y	CGGGGACAAAGTGGGCGTAGCATACATAATTGAAACTTGTGG	GCTACGCCCACTTTGTCCCCG
S102T	CGGGGACAAAGTGGGCGTAGCAACCATAATTGAAACTTGTGG	GCTACGCCCACTTTGTCCCCG
S102D	CGGGGACAAAGTGGGCGTAGCAGACATAATTGAAACTTGTGG	GCTACGCCCACTTTGTCCCCG
T166S	GCAGGAATCTCGGCTTATAGTCCC	GGGACTATAAGCCGAGATTCCTGC
T166V	GCAGGAATCGTGGCTTATAGTCCC	GGGACTATAAGCCACGATTCCTGC
F67V	CAAGCTATCCTTATGTTTTAGGGC	GCCCTAAAACATAAGGATAGCTTGTAACTCCAAATTTGTTTTTGC
THAS1 loop swaps		
loop1 swap	CACAAACAAATTTGGGATGACAAAGTATCCTTATGTTTTAGGGC	CTTTGTCATCCCAAATTTGTTTGTGCTCATTTCCCTGTCATATTGG
loop2 swap	GATGGCCATTTTGGAAATAATTTCGGGGCATGTTCAAATATAGC	GAAATTATTTCCAAAATGGCCATCTATTGATCCTGCTTCTGGACAG
HYS loop swaps		
loop1 swap	AAGAACAAATTTGGATTTACAAGCTATCCTTTTGTTATAGGGC	GCTTGTAAATCCAAATTTGTTCTTGCTCATTTCCAAGTCATAGTTGC
loop2 swap	GAATCAACAGACAGCAATTACGGTGGATGTTGTAATATAATGG	CATCCACCGTAATTGCTGTCTGTTGATTCTACTTTTGAACAG

The mutated codons are underlined.

Supplementary Table 6. List of THAS1 and HYS mutants that were screened for heteroyohimbine synthase activity against strictosidine aglycone. The experimental conditions of enzyme assays and the analytical conditions are reported in the Methods. The total conversion refers to the yield of reduction compared to the starting concentration of strictosidine aglycone. The measured concentrations of the three identified heteroroyohimbines (ajmalicine, mayumbine/19-epi-ajmalicine, tetrahydroasltonine) define the product ratio. The activity of all enzymes was compared to activity of the corresponding wild type.

Mutants	Total conversion	Product ratio Ajmalicine	Product ratio Mayumbine/19-epi-ajmalicine	Product ratio Tetrahydroal-stonine	Conversion compared to wild type
THAS1 Y56A	2.26%	3.57%	4.79%	91.64%	11.95%
THAS1 Y56E	0.05%	0.00%	29.09%	70.91%	0.28%
THAS1 Y56T	1.01%	7.62%	7.99%	84.39%	5.34%
THAS1 Y56S	4.13%	2.20%	5.91%	91.88%	21.86%
THAS1 Y56F	0.14%	54.17%	11.11%	34.72%	0.72%
THAS1 E59A	21.39%	4.14%	11.01%	84.84%	113.16%
THAS1 S102A	12.34%	0.95%	2.01%	97.04%	65.27%
THAS1 S102Y	5.50%	2.13%	2.10%	95.77%	29.12%
THAS1 S102T	12.91%	1.71%	1.50%	96.79%	68.31%
THAS1 S102D	0.54%	13.73%	3.87%	82.39%	2.84%
THAS1 T166S	0.64%	12.69%	5.26%	82.05%	3.39%
THAS1 T166V	0.30%	24.20%	7.14%	68.66%	1.61%
THAS1 F67V	18.84%	6.34%	6.34%	87.32%	99.69%
THAS1 loop1 swap	11.22%	3.86%	4.68%	91.47%	59.38%
THAS1 loop2 swap	0.39%	19.27%	9.02%	71.71%	2.05%
THAS1 loop1 + loop2 swap	0.64%	12.02%	7.86%	80.12%	3.37%
HYS loop1 swap	1.97%	26.29%	33.07%	40.63%	9.05%
HYS loop2 swap	9.34%	1.54%	5.19%	93.28%	42.83%
HYS loop1 + loop2 swap	0.70%	20.05%	58.22%	21.73%	3.22%
HYS H127A	0.86%	3.24%	3.53%	93.22%	3.92%
HYS H127N	2.46%	1.05%	0.77%	98.19%	11.27%
HYS F128A	0.08%	34.27%	8.29%	57.45%	0.38%
HYS F128Y	0.57%	47.95%	5.76%	46.29%	2.62%

Supplementary Table 7. List of primer pairs for MDR candidates for VIGS.

Medium Chain Reductases	Forward primer	Reverse primer
THAS1 Cr024553	GGCGCGAUGAGGTAACTGAAGTTGGCAGC	GGTTGCGATTCCTCCTAGACCGGCTATG
THAS2 Cr021691	GGCGCGAUGTAATGAAGGTCTTGAACCTTATTGTC	GGTTGCGAUCTTGCTTCTTGCTAATAGATGTACTAATTACAGT
THAS3 Cr010119	GGCGCGAUGTAGGCGTTAGTACTTACATTCGAACA	GGTTGCGAUTGGACTATAAGGAACTGTACCAGCA
THAS4 Cr032583a<br>HYS Cr032583b	GGCGCGAUCTACTTACAGTCCCTTGAGACGATATG	GGTTGCGAUTGGCACAATGGAGTGAGCCACTGGC

Supplementary Table 8. List of primer pairs for MDR candidates for qRT-PCR

Medium Chain Reductases	Forward primer	Reverse primer
THAS1 Cr024553	TCTTGGGAAGCTTATCATTTTAGG	AGCAGTACTGGCAGCC
THAS2 Cr021691	TGAAGGGACGTTTGTTATGC	GCTTATACTGCCAACGAACT
THAS3 Cr010119	CGATGGTACCCTTCTTATGC	TCCAGCACTTCCCACC
THAS4 Cr032583a<br>HYS Cr032583b	TTAGGGGTACCAGAGGAG	TGTGTTTCCTTCATACTTCCG

References

1 Krissinel, E. & Henrick, K. Secondary-structure matching (SSM), a new tool for fast protein structure alignment in three dimensions. *Acta Crystallogr D Biol Crystallogr* **60**, 2256-2268 (2004).

2 Qu, Y. *et al.* Completion of the seven-step pathway from tabersonine to the anticancer drug precursor vindoline and its assembly in yeast. *Proc. Natl. Acad. Sci. USA* **112**, 6224-6229 (2015).
